# Genomic epidemiology of coxsackievirus A24 variant during the 2024 acute hemorrhagic conjunctivitis outbreak in Coastal Kenya

**DOI:** 10.1371/journal.ppat.1014589

**Published:** 2026-09-15

**Authors:** John Mwita Morobe, Edidah M. Ong’era, Arnold W. Lambisia, Samuel O. Odoyo, Martin Mutunga, Esther N. Katama, Robinson Cheruiyot, Joyce U. Nyiro, Everlyn Kamau, Charlotte J. Houldcroft, Matt Keeling, Katherine Gallagher, Edward C. Holmes, Charles N. Agoti

**Affiliations:** 1 Kenya Medical Research Institute (KEMRI)-Wellcome Trust Research Programme, Kilifi, Kenya; 2 The Open University, Milton Keynes, United Kingdom; 3 University of California, San Francisco, United States of America; 4 Department of Genetics, University of Cambridge, Cambridge, United Kingdom; 5 The Zeeman Institute for Systems Biology and Infectious Disease Epidemiology, Research (SBIDER), University of Warwick, Coventry, United Kingdom; 6 Department of Infectious Diseases Epidemiology, London School of Hygiene and Tropical Medicine, London, United Kingdom; 7 School of Medical Sciences, The University of Sydney, Sydney, New South Wales, Australia; 8 School of Public Health, Pwani University, Kilifi, Kenya; University of North Carolina at Chapel Hill, UNITED STATES OF AMERICA

## Abstract

Several African countries experienced a surge in acute hemorrhagic conjunctivitis (AHC) cases in 2024. Investigations in Kenya, Mayotte (an Indian Ocean island) and Tanzania identified coxsackievirus A24 variant (CVA24v) as the causative agent. To date, however, limited genomic data exist to elucidate the sources, epidemiology, and evolution of CVA24v in Africa. We generated 245 CVA24v genomes from samples collected between January and September 2024 in coastal Kenya, representing the largest outbreak CVA24v genomic data set available globally. Phylogenetic analysis showed that these viruses belonged to genotype IV, falling into two major clusters that differed by 52 nucleotide and five amino acid changes, and with an intra-species recombination event involving another enterovirus in the 3D^*pol*^ gene. Notably, the Kenyan sequences clustered closely with contemporaneous Africa (2024) sequences, specifically Mayotte and Malawi, reflecting a regionally connected CVA24v outbreak, but were distinct from those sampled previously in Asia in 2023, with phylodynamic analysis revealing that the Most Recent Common Ancestor of Kenyan sequences existed between June and October 2023. In summary, this study provides the first detailed genomic analysis of CVA24v from Africa to inform future surveillance and control strategies.

## Author summary

Acute hemorrhagic conjunctivitis (AHC) is a highly contagious eye disease that can cause explosive outbreaks and widespread morbidity, yet its viral drivers remain poorly understood in many regions, especially Africa. In 2024, coastal Kenya experienced a major AHC outbreak caused by coxsackievirus A24 variant (CVA24v). Using samples collected through routine disease surveillance and outbreak response activities, we generated the largest collection of CVA24v genomes from a single African setting. Our analyses show that the 2024 Kenyan outbreak was driven by two closely related viral lineages within genotype IV that have a history of recent intra-species recombination and were linked to viruses circulating in Eastern and Southern Africa and nearby Indian Ocean islands. These viruses were genetically different from those causing outbreaks in Asia in the preceding months, despite both regions experiencing near concurrent outbreaks, suggesting that other factors such as waning population immunity and favorable environmental conditions may play a role in driving new epidemics. Phylogenetic and phylodynamic analyses revealed an epidemiological pattern of short, intense epidemic waves separated by extended periods of low-level undetected transmission. Together, these findings highlight the importance of sustained genomic surveillance to understand CVA24v transmission, evolution, and outbreak emergence.

## Introduction

Coxsackievirus A24 variant (CVA24v) is a genetically and epidemiologically distinct lineage of coxsackievirus A24 (CVA24) within the species *Enterovirus coxsackiepol* (formerly *Enterovirus C* or EV-C) (family *Picornaviridae,* genus *Enterovirus*), and a leading cause of acute hemorrhagic conjunctivitis (AHC) [1–4]. The prototype CVA24 strain was first isolated in South Africa in 1951 [5], but was not associated with disease in humans. A pathogenic variant of this virus, denoted CVA24 variant (CVA24v), emerged in Singapore in 1970, where it caused a large AHC outbreak. Since its emergence, nearly all documented large-scale AHC epidemics have been attributed to CVA24v, while classical CVA24 sequences are now rarely reported in contemporary data sets. Indeed, CVA24v has been implicated in AHC outbreaks in Asia [3,4,6–8], Africa [9–12], and South America [13,14].

CVA24v infection is characterized by the rapid onset of ocular pain, sub-conjunctival hemorrhage, swollen eyelids, foreign body sensation, epiphora, eye discharge and photophobia [15–17]. Typically, the symptoms follow an incubation period of 12–48 hours and persist for three to five days before completely resolving (10–14 days) [16,18]. CVA24v infection is detectable in respiratory, saliva, stool and ocular samples [19–21]. Outbreaks drive substantial morbidity, productivity losses, and work/school absenteeism, increased visits to eye clinics, and often lead to unnecessary antibiotic use, contributing to antimicrobial resistance concerns [22–24].

The CVA24v genome comprises a positive-sense, single-stranded RNA molecule of approximately 7.4 kilobases [13]. The genome contains a single long open reading frame (ORF) that encodes a polyprotein, which is then proteolytically processed into four structural proteins (VP1–VP4) and seven non-structural proteins (2A–2C and 3A–3D) involved in viral replication and virus–host interactions [25]. The genetic diversity within the VP1 region has been widely used for molecular epidemiological classification of CVA24v into eight distinct genotypes (GI–GVIII) [26].

CVA24v-associated AHC outbreaks often affect thousands to hundreds of thousands of people within a short period [27]. These outbreaks re-emerge at variable intervals [27], typically 3–5 years apart [27], with large-scale epidemics occurring at approximately 8–10 years intervals in tropical and subtropical countries [27]. Yet how CVA24v persists between outbreaks remains unclear. The re-emergence of CVA24v in communities may be in part due to antigenic evolution in the viral capsid proteins (VP), as well as waning population immunity, both of which can facilitate reinfection [13,28,29]. Neutralizing antibody immune responses have also been found to decline significantly within one-year post-infection [30].

Detailed molecular epidemiological investigations of CVA24v in Africa remain scarce, limiting our understanding of its underlying evolutionary and transmission dynamics. In Kenya, for example, the characterisation of previous outbreaks has been limited to case studies or outbreak reports [31]. In early 2024, a CVA24v outbreak was observed along the Kenyan coast [32,33], coinciding with contemporaneous outbreaks from several other African countries including Tanzania, Rwanda, Burundi, Malawi, Uganda [9,10,34] Madagascar and Mayotte [11,12]. Shortly before this, CVA24v outbreaks were reported across parts of Asia in 2023, including in Pakistan, China, India, Nepal, and Bhutan [1,3,6,7]. The geographic and temporal overlap or succession of these outbreaks suggests epidemiological linkage. However, detailed CVA24v genomic analyses to elucidate virus origins, phylogenetic relationships, and transmission pathways are lacking.

Herein, we analyzed respiratory and stool samples collected through ongoing acute respiratory infection and acute diarrheal disease surveillance studies in Kilifi County, coastal Kenya (Fig A in S1 Appendix), alongside ocular samples collected during the AHC outbreak in 2024 in Mombasa County [32,33]. A subset of positive samples underwent whole-genome sequencing (WGS) followed by phylogenetic and phylodynamic analysis incorporating all publicly available CVA24v whole genome sequences (n = 159, as of November 1, 2025). In addition, 418 CVA24v partial VP1 sequences were used to assess clustering patterns and classification among the CVA24v genotype IV (G-IV) sequences. This work builds on prior Kenyan [32,33] and regional studies [9,12], which have largely focused on descriptive epidemiology of previous CVA24v detections. In doing so, we provide the first comprehensive genomic analysis of a CVA24v outbreak in an African setting, detailing its genetic diversity, evolutionary patterns, and local transmission dynamics.

## Results

### CVA24v detections in Kilifi

**Fig 1:**
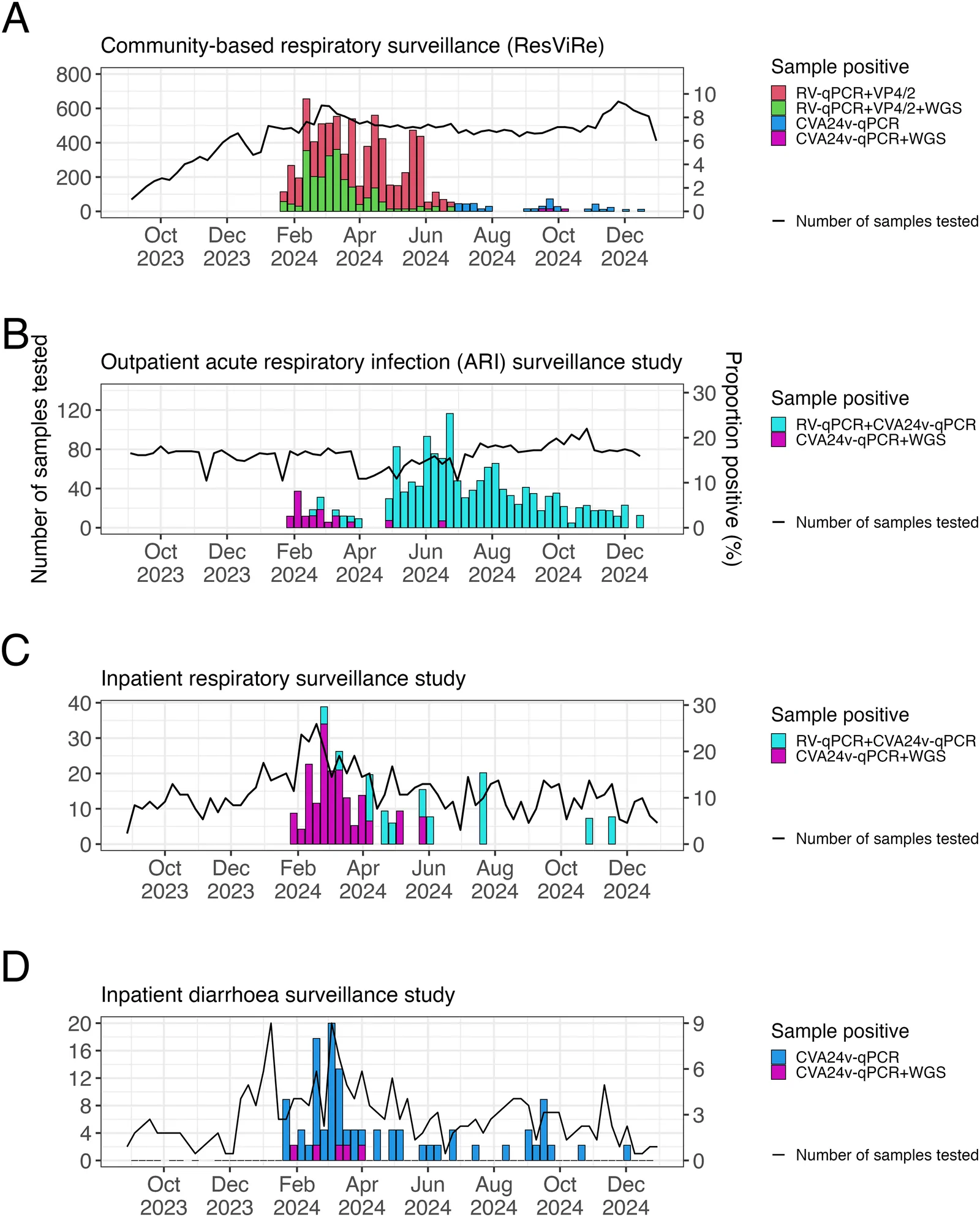
Temporal distribution of CVA24v positivity across the four surveillance platforms. **(A)** Temporal distribution of CVA24v positives at the community-based respiratory surveillance. **(B)** Temporal distribution of CVA24v positives in the in-patient pneumonia surveillance. **(C)** Temporal distribution of CVA24v positives in the out-patient acute respiratory infection surveillance. **(D)** Temporal distribution of CVA24v positives in the in-patient diarrheal etiology surveillance. The bar plot shows the percentage of samples positive for CVA24v, stratified by the detection or confirmatory methods used, while the black line represents the total number of samples tested.

The distribution of samples collected, tested, CVA24v positive, and sequenced across our Kilifi surveillance platforms is summarized in Table 1. The analysis identified the first CVA24v positive sample from a ResViRe (Respiratory Virus Reinfections) study participant on January 23, 2024. Thereafter, a rapid increase in CVA24v detections was observed in the other surveillance platforms (Fig 1). Peak CVA24v positivity varied by study, with the earliest peak recorded in the inpatient diarrhoea study in epidemiological week 5 (11.1%), followed by the outpatient health facility (HF) study in week 6 (8.1%), the ResViRe study in week 7 (9.6%), and the paediatric inpatient pneumonia (IP) study in epidemiological week 9 (29.2%) (Fig 1). Following this, case numbers declined steadily until June 2024, with sporadic detections up to December 2024 (Fig 1).

**Table 1:** Distribution of samples collected from acute haemorrhagic conjunctivitis (AHC) outbreak in Mombasa, and the various acute respiratory or diarrhoea surveillance platforms in the Kilifi Health and Demographic Surveillance System (KHDSS) between August 2023 and December 2024, indicating the total number of samples collected and tested using various diagnostic approaches, positive for CVA24v and successfully sequenced.

Surveillance Platform	Period	Number of Samples Collected and Tested for RV	RV Positive by qPCR^$^	CVA24v Positive Samples by qPCR^&^	CVA24v Positive Samples by VP4/2 Sequencing^£^	Samples Sequenced (WGS)
Community-based homestead surveillance study (ResViRe study) ‡	31-Aug-2023 – 31-Dec-2024	31,297	2,742	36	563	182
Pediatric in-patient pneumonia surveillance	01-Aug-2023 – 31-Dec-2024	1,034	182	48	0	33
Health facility out-patient surveillance	01-Jan-2024 – 31-May-2024	4,965	750	66	0	22
In-patient diarrhea etiology surveillance	01-Jan-2023 – 31-Dec 2024	434	–	64	0	5
Mombasa AHC outbreak*	15 Feb 2024	15	–	–	3	3
Total	**–**	**37,745**	**3,674**	**214**	**566**	**245**

‡In the ResViRe study, samples were collected from 713 individuals, of whom 218 (26.7%) tested positive for CVA24v. Of these,182 genome sequences were recovered, representing samples from 78 individuals.

*CVA24v identified using metagenomic sequencing.

$ RV (rhinovirus) identified using RV-specific quantitative reverse transcription polymerase chain reaction (qPCR) assay.

& CVA24v identified using a RV qPCR assay followed by typing using VP4–VP2 sequencing and/or by CVA24v-specific qPCR.

^£^CVA24v identified using a CVA24v-specific qPCR assay.

CVA24v-positive individuals were younger in the ResViRe study (median age 5 vs 19 years in negative individuals; p < 0.001), with over half (51.3%) of infections detected in 0–5 years (Table A in S1 Appendix). Similarly, CVA24v-positive participants were younger (median age 9 years vs 16 years; p = 0.004) in the out-patient surveillance, with children <5 years comprising 45.4% of positives compared to 29.2% of negatives. Detections in paediatric in-patient pneumonia and diarrheal admissions were more frequent in infants (0–11 months). Significant differences were observed in positivity across administrative locations, with the Roka and Zowerani areas (p = 0.015) showing high positivity rates in the ResViRe study, and prevalence similarly varied by health facility (p = 0.042) in the HF study, being highest in Pingilikani and Mavueni outpatient clinics. Positivity across by gender was comparable in all surveillance platforms (Table A in S1 Appendix).

### CVA24v sequencing and genotyping

Whole-genome sequencing was attempted on 333 CVA24v-positive samples collected between 23^rd^ January and 23^rd^ September 2024. Priority was given to samples with a quantitative reverse transcription polymerase chain reaction (qPCR) cycle threshold (Ct) value <30.0 for better genome recovery or yields [35]. Additionally, some samples were excluded after sequencing revealed assay cross-reactivity with other enteroviruses, such as Coxsackievirus A13 (CVA13) and Enterovirus-C99 (EV-C99). A total of 245 (74.0%) samples yielded complete or near-complete genome sequences (coverage >98.0%). These sequences ranged from 7,304–7,430 nucleotides (nt) in length and comprised a 5′ untranslated region (UTR) of ~750 nt, a 3′ UTR of ~69 nt, and a single open reading frame (ORF) of 6,645 nt.

Of the recovered 245 genome sequences, 182 (74.3%) were from 78 individuals enrolled in the community-based homestead surveillance platform (ResViRe study), of whom 43 contributed more than one sequence. For the remainder, 33 (13.5%) were from the in-patient pneumonia surveillance, 22 (9.0%) from the out-patient ARI surveillance, 5 (2.0%) from the diarrhoea aetiology surveillance and 3 (1.2%) from the clinically confirmed AHC outbreak in Mombasa County (Table 1). Both phylogenetic analysis and the Enterovirus Genotyping Tool (based on partial viral protein 1 (VP1) sequence) [36] determined that all the 245 Kenyan sequences belonged to genotype IV.

### Global context of the Kenya CVA24v strains

**Fig 2:**
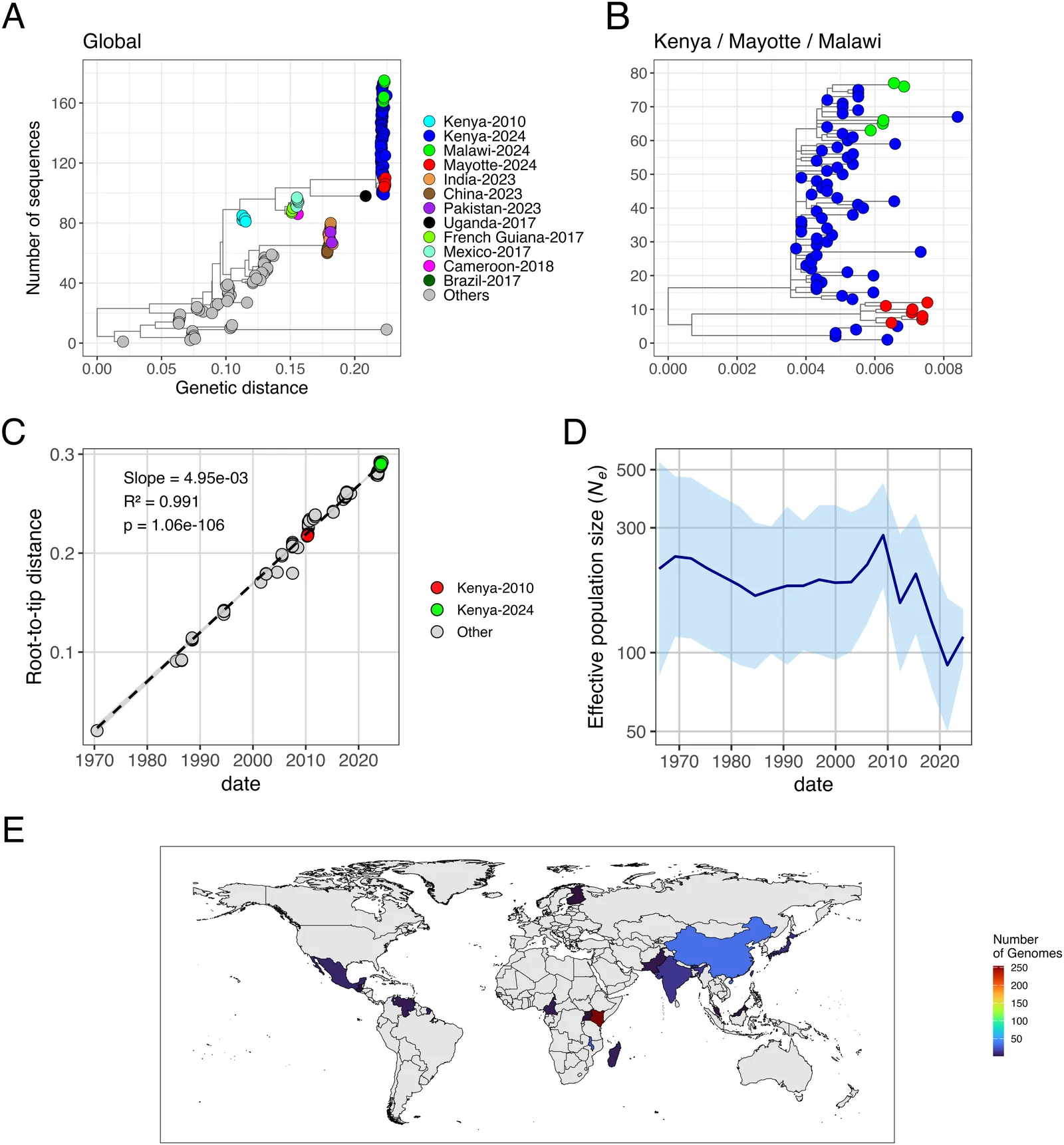
Genetic diversity, root-to-tip divergence, and demographic reconstruction of global CVA24v strains sampled between 1970 and 2024 (A) ML phylogenetic tree of 194 whole-genome sequences selected from the global (129) and Kenya (n = 65) data sets. **(B)** ML phylogenetic tree of 80 whole-genome sequences from Kenya, Mayotte and Malawi. Tip labels are colored by country of sampling. **(C)** Root-to-tip regression showing accumulation of genetic divergence in CVA24v over time. The phylogeny was rooted using TreeTime. **(D)** ML skyline reconstruction of genetic diversity (presented as the effective population size, *Ne*) of CVA24v over time, assuming 50 generations per year. The solid line represents the median estimate of *Ne*, while the shaded area shows the approximate 95% confidence bounds (±2 standard deviations of the log-likelihood). **(E)** World map showing the distribution of countries that have deposited CVA24v WGS in GenBank as of 01 November 2025. Color intensity indicates the number of sequences contributed by each country (using a standard “hotter” scale for higher density). The map was generated in R. The base layer was obtained from the Humanitarian Data Exchange (Global Administrative Boundaries – OSM), https://data.humdata.org/dataset/global-international-boundaries-osm. The data set is distributed under the Creative Commons Attribution 4.0 (CC BY 4.0) license, permitting broad use including commercial, non-commercial, and academic applications.

**Fig 3:**
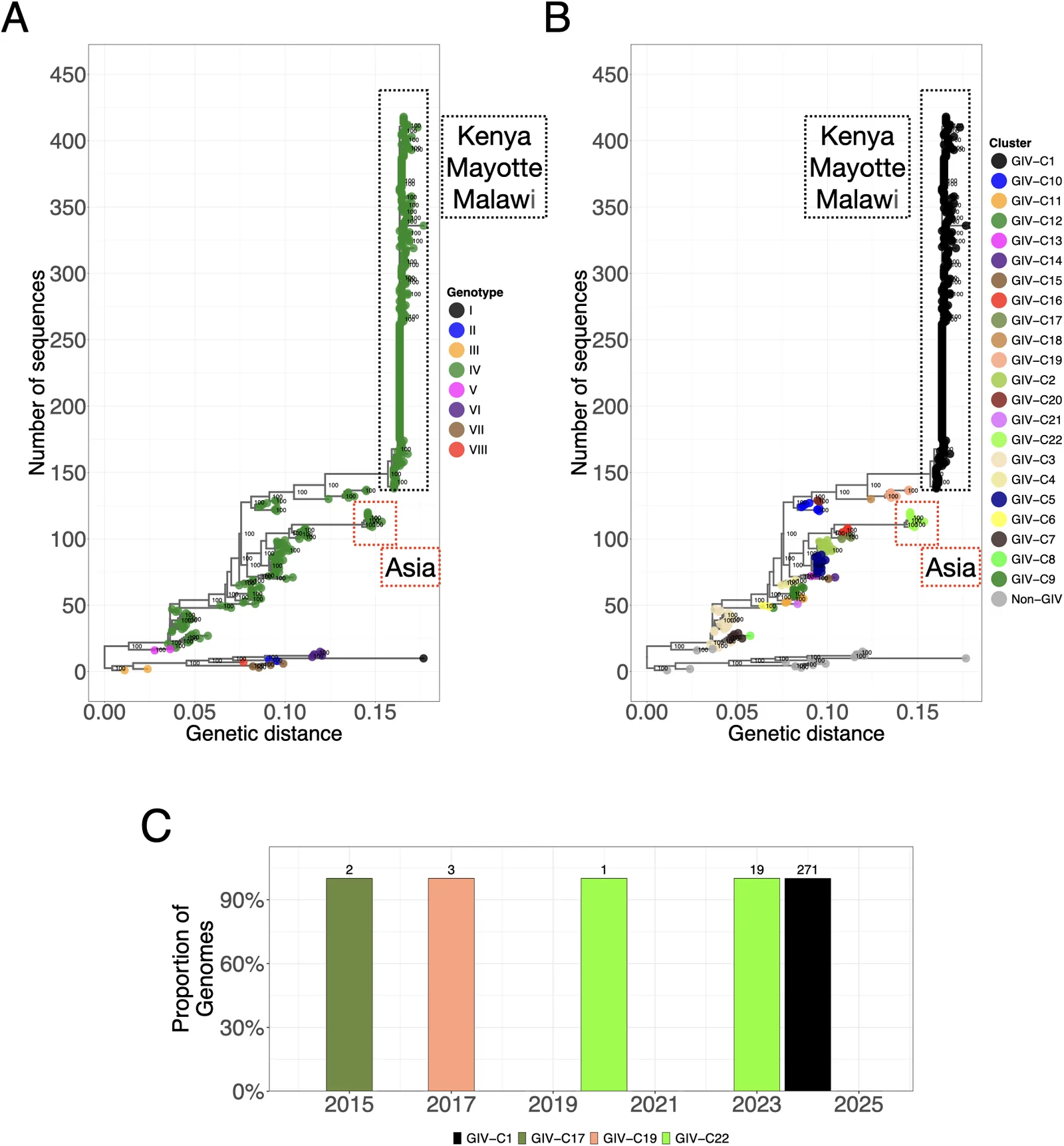
Genetic diversity of globally circulating CVA24v. **(A)** ML phylogenetic tree of global CVA24v VP1 sequences sampled between 1970 and 2024, showing the clustering of major CVA24v genotypes. **(B)** ML phylogenetic tree of genotype IV CVA24v partial VP1 sequences, showing clustering into distinct sub-groups (GIV-C1 to GIV-C22) using a 0.05 nucleotide divergence threshold. The black dashed box indicate the East African sequences while the red dashed box indicates sequences responsible for the Asia outbreak. **(C)** Temporal distribution of CVA24v sub-groups detected between 2015–2024, classified using the established 0.05 VP1 pairwise divergence threshold for genotype IV (GIV) sub-group delineation.

Global phylogenetic analysis of whole-genome sequences revealed that CVA24v viruses from the same continent and outbreak often formed a monophyletic group (Fig 2A and Fig B in S1 Appendix). The observed diversity within genotype IV warranted additional analyses to fully characterize recent global CVA24v circulation. On applying a clustering analysis to the partial VP1 coding region of genotype IV strains (including global sequences (n = 418) and those generated in this study) using VSEARCH v2.30.2 [37], sequences were deduplicated and clustered with the --cluster_fast algorithm at a 95% nucleotide identity threshold (i.e., 0.05 divergence). This threshold was chosen because it produced well-defined clusters consistently supported by high bootstrap values (>70%) [38]. This analysis placed the genotype IV sequences into 22 distinct sub-groups, herein denoted GIV-C1–C22 (Fig 3A and 3B). Temporal analysis of VP1-defined sub-groups revealed shifts in the circulation and predominance of distinct CVA24v lineages between 2015 and 2024 (Fig 3C).

Notably, all the 2024 Kenyan and other Eastern/Southern Africa sequences fell into the same sub-group (GIV-C1), but formed three distinct clusters within this subgroup, herein denoted C1/1/24, C1/2/24, and C1/3/24, two of which (C1/1/24 and C1/2/24) were present in Kenya (Fig B in S1 Appendix). Of particular importance was that the 2024 Kenyan, Malawi, and Mayotte sequences belonged to a different sub-group from the strains associated with outbreaks in India, Nepal, Bhutan, China, and Pakistan in 2023, even though they are close to the Kenyan outbreak in time (Fig 2A and Fig B in S1 Appendix). Rather, the Kenyan sequences were more closely related to sequences from the 2017–2018 CVA24v outbreaks in Uganda, French Guiana, Brazil, Cameroon, and Mexico, strongly suggesting that the 2024 Kenya outbreak was unlikely to have been imported from Asia (Fig 2A and Fig B in S1 Appendix).

### Timing of the global CVA24v common ancestor and changes in effective population size

The global CVA24v whole-genome data set showed a strong molecular clock signal from our root-to-tip regression analysis on the ML phylogeny (R^2^ = 0.991) (Fig 2C). To account for the potential impact of recombination on molecular clock analysis, we masked the 3D ^*pol*^ recombinant region (nt ~ 6,400–7,400; see below) before the phylodynamic analysis. Accordingly, we were able to estimate the mean evolutionary rate of the global sequences to be 4.95 × 10^-3^ nucleotide substitutions per site per year (95% HPD: 4.71 × 10^-3^ – 5.11 x 10^-3^ (Fig 2C). Based on this rate, the Time of the Most Recent Common Ancestor (tMRCA) for the global CVA24v was estimated to be between June 1965 – February 1967 (95% HPD; mean date of April 1966). A subsequent analysis of the global demographic history of CVA24v using a skyline coalescent model revealed marked variation in genetic diversity (reflected in effective population size (*Ne*) over time) that corresponded to outbreaks in different global regions: 1990–2000 in Asia, 2000–2010 in Asia and Africa, and 2015–2020 in South America, Africa and Asia (Fig 2D). A sharp increase in *Ne* was observed from between 2022 and 2024, coinciding with the recent outbreaks in Africa and Asia (Fig 2D).

### Genetic diversity of Kenya and other Africa CVA24v strains

**Fig 4:**
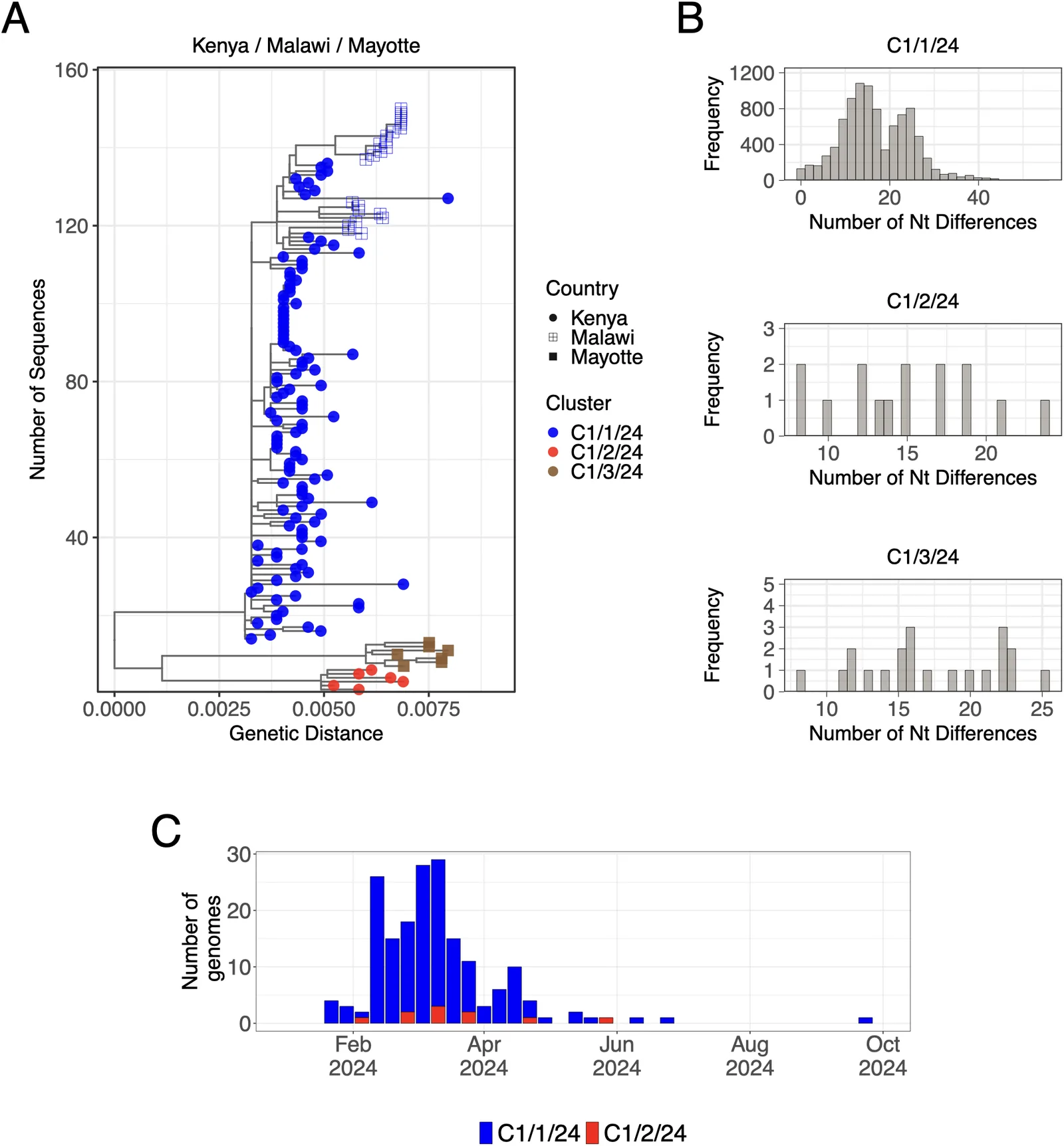
Genetic diversity of Kenya, Malawi and Mayotte CVA24v sequences. **(A)** ML phylogenetic tree of the Kenya 2024 CVA24v whole-genome sequences. Tips are colored according to the major clade identified (C1/1/24 in blue, C1/2/24 in red and C1/3/24 in tan). Different shapes denote the country where the virus was sampled. **(B)** Pairwise SNP-distance distribution plots of C1/1/24, C1/2/24 and C1/3/23 sequences. **(C)** Temporal distribution of the C1/1/24 and C1/2/24 clusters in coastal Kenya.

Next, we focused on the local dynamics of the Africa 2024 CVA24v outbreak using whole-genome sequences. The C1/1/24 cluster predominantly comprised Kenyan genomes with interspersed Malawi sequences, the C1/2/24 cluster exclusively comprised sequences from Kenya, while the C1/3/23 cluster only contained sequences from the Indian Ocean island, French Overseas Department of Mayotte (Fig 4A). Within C1/1/24 and C1/2/24, multiple well-supported sub-clusters (bootstrap support >70%) were observed, which frequently included viruses from the same location or surveillance platform (Fig C in S1 Appendix). The mean evolutionary rate of the 2024 Kenya genomes was estimated at 5.351 × 10 ^-3^ nucleotide substitutions/site/year (95% HPD interval: 3.914 × 10 ^-3^ – 6.793 × 10 ^-3^) and the estimated tMRCA was around September 2023 (95% HPD interval: June 2023 – October 2023). The nucleotide sequence similarities between C1/1/24, C1/2/24 and C1/3/24 ranged from 98.42% to 100% with amino acid similarities from 99.99% to 100%, while the pairwise single nucleotide polymorphism (SNP) differences among sequences in the three clusters combined ranged from 0 to 101. Within the C1/1/24 cluster, SNP distances ranged from 0 to 52, in C1/2/24 they ranged from 8 to 33, while in cluster C1/3/24 they ranged from 8 to 25 (median: 14) (Fig 4B). The two clusters (C1/1/24 and C1/2/24) identified in Kenya co-circulated throughout the CVA24v outbreak (Fig 4D).

### Analysis of amino acid variation and selection analysis

**Fig 5:**
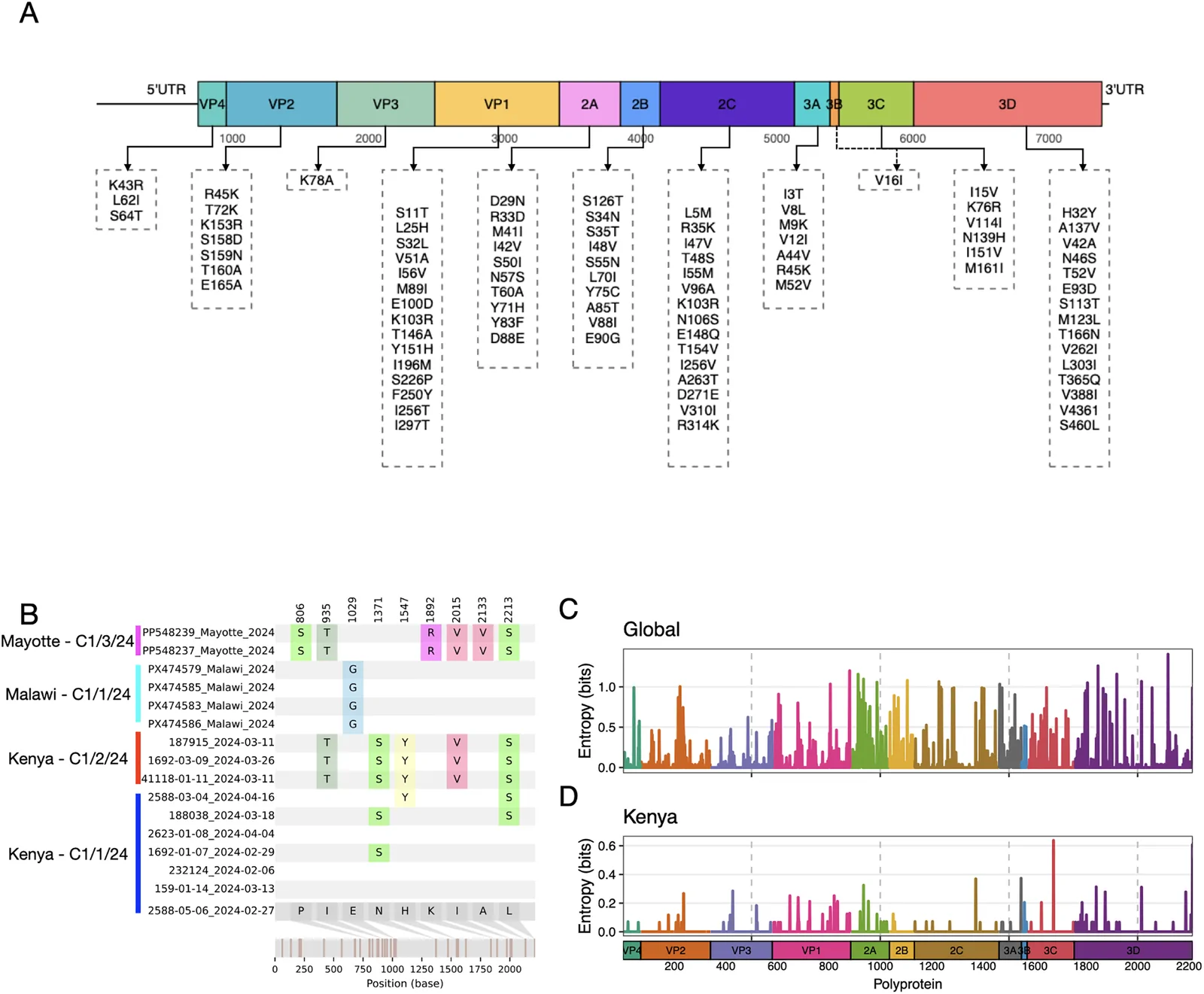
Amino acid variability across the CVA24v genome. **(A)** Summary of amino acid changes across different genomic regions of the Kenya CVA24v, comparing the prototype strain (D90457.1/Singapore/1970). **(B)** Amino acid differences between sequences from Kenya, Malawi, and Mayotte, with the Kenyan sequence acting as the reference. **(C)** Global sequences showing variable amino acid positions and their distribution across coding regions. **(D)** Variable amino acid positions in the Kenyan sequences and their distribution across coding regions. Peaks represent regions with higher amino acid variability.

The 2024 Kenya CVA24v whole-genome sequences were compared to the prototype strain EH24/70 (GenBank accession no. D90457); this revealed amino acid substitution at 89–95 codon sites (Fig 5A), with 41–48 amino acid differences compared to the earliest genotype IV genomes. Most substitutions occurred in the VP1, 2C, and 3D^*pol*^ gene, with each exhibiting substitutions at 15 codon sites (Fig 5A). The C1/1/24 and C1/2/24 clusters were distinguished by five amino acid substitutions at codon sites 935 (I/T), 1371 (N/S), 1547 (H/Y), 2015(I/V) and 2213(L/S) (Fig 5B). Kenya 2024 CVA24v sequences had one amino acid difference relative to Malawi strains and five when compared to Mayotte sequences (Fig 5B), but diverged at 51 codon sites from 2023 Asian strains. The Kenyan 2024 CVA24v sequences differed at 27 sites from the Kenya 2010 CVA24v sequences (Fig D in S1 Appendix). Entropy analysis of the global CVA24v data set revealed substantial heterogeneity throughout the polyprotein, which was mirrored in the Kenya-only data set (Fig 5C and D). Outside the protein coding region, the Kenyan strains exhibited a single-nucleotide insertion at position 102, a single-nucleotide deletion at position 585, and a four-nucleotide deletion spanning positions 117–120 within the 5′ untranslated region (UTR) relative to the prototype strain EH24/70.

Analyses of selection pressures were conducted using three methods available in HyPhy algorithm: Single Likelihood Ancestor Counting (SLAC), Fixed Effects Likelihood (FEL), and Fast, Unconstrained Bayesian Approximation (FUBAR). SLAC estimated an overall mean ratio of non-synonymous to synonymous substitutions (i.e., d_N_/d_S_) of 0.0706, indicative of strong purifying selection. Despite this, the three approaches (SLAC, FEL, and FUBAR) collectively identified evidence of positive selection at 10 codon positions (Table 2). Under SLAC, five codons – VP2 (234, 236), VP1 (683, 881), (2C) 1371 and (3C) 1601 – showed evidence of increased fixation of non-synonymous mutations (d_N_/d_S_ > 1). The FEL method identified 10 codon positions subject to positive selection: VP2 (234, 236), VP1 (683, 830, 871, 881), 2C (1371), 3A (1506), 3C (1601), 3D (2124). FUBAR also identified six codon positions under pervasive positive selection with posterior probabilities ≥ 0.9: codons VP1 (683, 830, 871, 881), 2C (1371), and 3C (1601) (Table 2).

**Table 2:** Codon sites inferred to be under positive selection in CVA24v using SLAC, FEL, and FUBAR.

Gene	Codon position	SLAC: dN–dS	SLAC p-value	FEL: α (dS)	FEL: β (dN)	FEL p-value	FUBAR: α	FUBAR: β	FUBAR posterior (β > α)
VP2	234	3.955	0.043	0	1.434	0.0164	–	–	–
VP2	236	3	0.088	0	0.996	0.0396	–	–	–
VP1	683	6.046	0.041	0.418	2.166	0.0598	0.994	6.782	0.948
VP1	830	–	–	0.642	2.562	0.0537	1.008	6.801	0.949
VP1	871	–	–	0.321	1.645	0.0735	0.843	5.278	0.933
VP1	881	5.794	0.055	0.311	2.887	0.0063	0.677	8.286	0.998
2C	1371	4.342	0.036	0	1.895	0.0029	0.441	6.448	0.995
3A	1506	–	–	0	1.07	0.0453	–	–	–
3C	1601	5.218	0.089	0.395	2.29	0.0461	0.828	6.422	0.959
3D^*pol*^	2124	–	–	0	0.809	0.0742	–	–	–

Codons were considered to be under strong positive selection if supported by at least two independent methods, defined as SLAC p < 0.05, FEL p < 0.05, or FUBAR posterior probability ≥ 0.9 for β > α.

### Recombination of CVA24v with other enteroviruses

**Fig 6:**
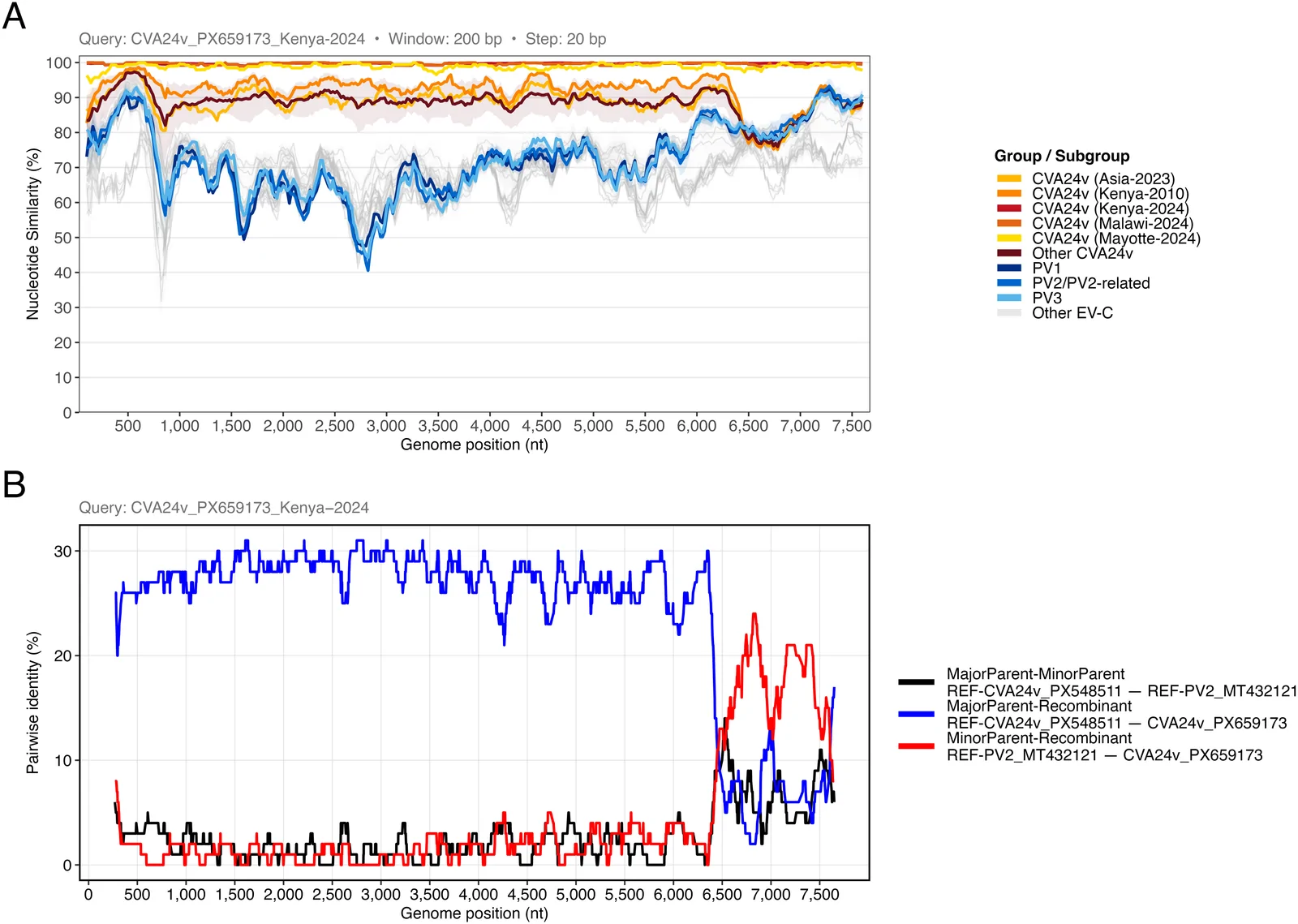
Nucleotide similarity between CVA24v and other species C enteroviruses. **(A)** Nucleotide similarity plots between Kenyan CVA24v genomes and other selected sequences from enterovirus C sequences obtained from GenBank as of 15th August 2025. The query sequence is one of the 245 from this study (accession number PX659173) and is compared with 2,418 *Enterovirus Coxsackiepol* sequences, including poliovirus (PV1, PV2, PV3) and non-polio EV-C reference sequences. **(B)** RDP5 BOOTSCAN analysis showing CVA24v as the major parent and PV2 as the closest possible minor parent. The analysis was based on a 200-nucleotide sliding window with a 20-nucleotide step size.

To detect possible recombination involving CVA24v, we conducted pairwise similarity analysis using a representative 2024 Kenyan CVA24v sequence as the query against an expanded global data set of 2,418 sequences, including poliovirus types 1, 2, and 3 (PV1–PV3) and a broad representation of non-polio EV-C sequences. This revealed that the 2024 Kenya/Mayotte/Malawi sequences shared > 98% nucleotide similarity across the genome (Fig 6A). In contrast, other global CVA24v sequences, predominantly from Asia and South America, exhibited 83–95% nucleotide similarity across most of the genome, with a marked drop to approximately 78% within the 6,400–7,200 nt region corresponding to the 3D^*pol*^ (Fig 6A). Polioviruses and other EV-C sequences displayed generally low nucleotide similarity (between 50–80%) to the Kenyan sequence, although relatively higher similarity was observed in the 5′ untranslated region (UTR) and, strikingly, in the 3D^*pol*^ region (6,400–7,200 nt). This elevated similarity in the 3D^*pol*^ region was observed across multiple EV-C species and is suggestive of intra-species recombination (Fig 6A).

**Fig 7:**
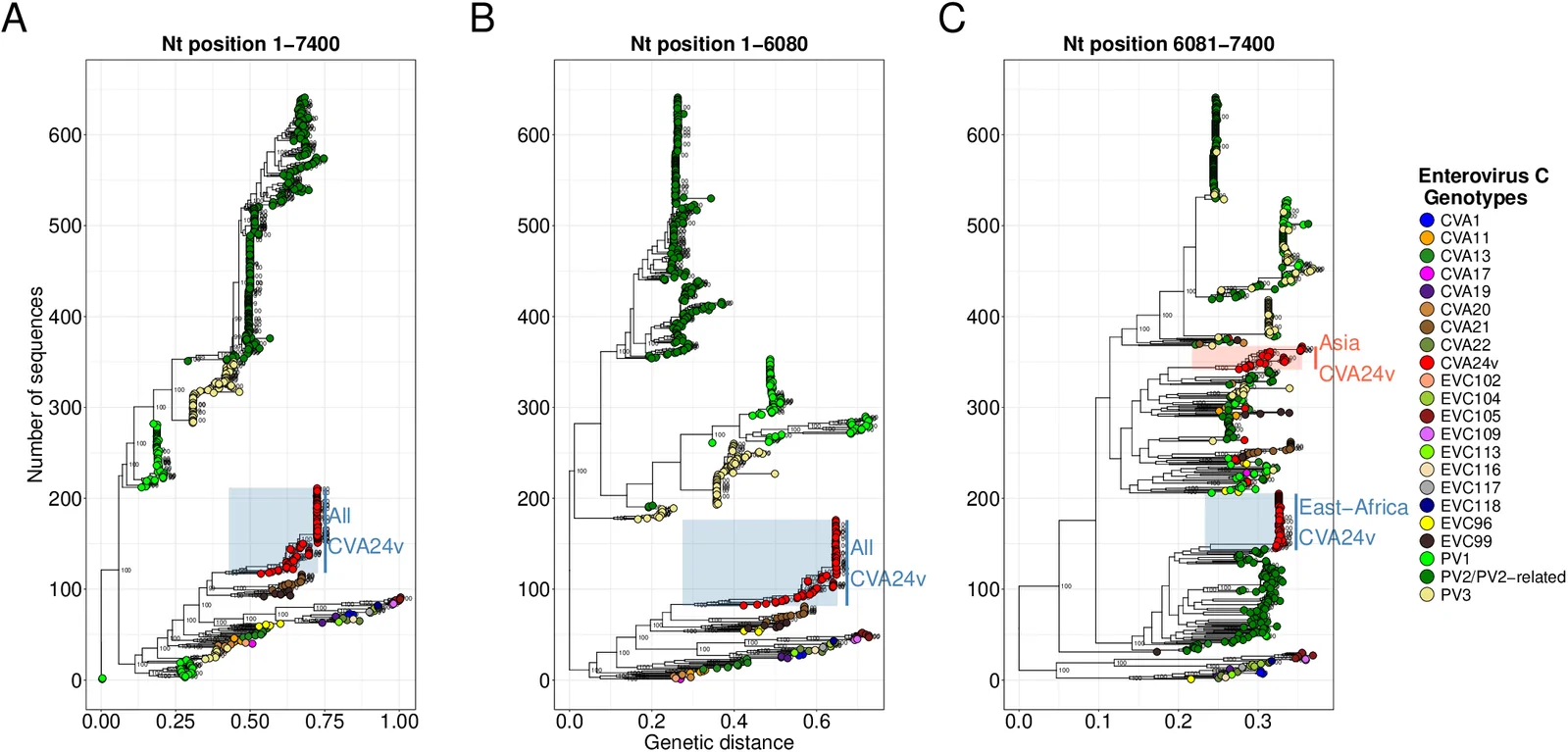
ML phylogenetic trees of CVA24v and other Enterovirus C (EV-C) genomes demonstrating the 3D^*pol*^ intra-species recombination event. **(A)** Phylogeny based on nucleotide positions 1–7400 (complete genome). **(B)** Phylogeny based on nucleotide positions 1–6080 (upstream of the inferred recombination breakpoint), showing that the 2024 East African CVA24v outbreak sequences cluster as a distinct monophyletic group within Genotype **IV. (C)** Phylogenetic based on nucleotide positions 6081–7400 (downstream of the inferred recombination breakpoint, corresponding to the 3D^*pol*^ region). A major topological incongruence is observed: the 2024 East African outbreak sequences and Uganda 2017 are phylogenetically distinct from other CVA24v lineages and instead cluster with recombinant West African PV2-related sequences. Bootstrap support values ≥99% are shown for internal nodes.

To further investigate potential recombination within this data set we utilised various methods implemented in the Recombination Detection Program (RDP v5.74). This analysis identified a putative intra-species recombination event in the 3D^*pol*^ region spanning position 6080 – 7341 (ungapped alignment coordinates; gapped equivalent 6418–7627 in the alignment). We then used ML phylogenetic analysis to identify the lineages involved in these putative recombination events and assess their topological support. For most of the viral genome (nt positions 1–6080) the CVA24v sequences formed a distinct and well-supported clade (Fig 7). In contrast, a differing topological pattern was seen in the region 6081–7400 that corresponds to the 3D^*pol*^ gene. Although relatively long branches and sampling gaps make the exact parental lineages for this challenging to identify, the Kenya/Mayotte/Malawi (2024) and Uganda (2017) sequences clustered (with relative strong support) with PV2-related sequences from West Africa (Fig 6B, Fig 7, Fig E in S1 Appendix). Importantly, these PV2-related sequences are themselves recombinant in the 3’ region and hence genetically distinct from the Sabin PV2 strain.

## Discussion

We leveraged clinical samples collected from ongoing respiratory/diarrheal disease surveillance studies within the Kilifi Health and Demographic Surveillance System (KHDSS) [39], Kilifi County, and during AHC outbreak response in Mombasa County [32,33], to generate the largest ever data set of CVA24v genomes from a single geographical location, both in Africa and globally, during an outbreak. Prior to this study, only 10 CVA24v genomes were available from Kenya on GenBank and 149 from the rest of the world. The newly generated sequence data provide valuable insights into CVA24v genomic epidemiology at the local scale, reveal evolutionary dynamics and putative intra-species recombination within Enterovirus C, and highlight regional epidemic inter-connectedness during the 2024 CVA24v outbreak in Eastern Africa.

In this study a CVA24v positive case was first detected in Kenya on January 23, 2024 through community-based homestead surveillance, after which a rapid surge in cases was observed in the other three disease surveillance platforms run by the Kenya Medical Research Institute–Wellcome Trust Research Programme (KEMRI-WTRP). We note that the design of these inpatient and outpatient surveillance platforms heavily targets children which inherently introduces sampling bias when assessing age-related burden. However, the community homestead-based surveillance and the outpatient HF surveillance that recruited patients across all age groups disproportionately reported infections in younger age groups. While healthcare-seeking behavior and study design limit our ability to make definitive claims regarding population level age burden, our findings strongly suggests CVA24v infection predominantly affected children during the 2024 outbreak. This pattern is consistent with the epidemiological profile of many enterovirus infections [40,41] and aligns with previous reports of CVA24v-associated AHC outbreaks [8,42].

The Kenya 2024 CVA24v sequences fell as two major clusters – C1/1/24 and C1/2/24 – within subgroup GIV-C1 of viral genotype IV. Similar patterns have been seen in previous CVA24v outbreaks, in which divergent clusters within genotype IV co-circulated during a single outbreak [38,43]. This pattern is indicative of *in situ* diversification driven by both multiple introductions and local spread [13,19,44]. We observed a close phylogenetic relationship between the 2024 Kenyan sequences and strains from Mayotte and Malawi, as well as earlier strains from Uganda, suggesting regional persistence of the virus for many years [9,12,32]. At the global scale, the 2023–2024 CVA24v outbreaks appear to be distinct by continent, with different genotype IV clades predominating different geographic regions. Indeed, the viral clade associated with the 2023 Asia AHC outbreaks (Bhutan, Pakistan, India, China and Nepal) [3,6,7,44] was distinct from that associated with the Kenya, Malawi, and the Indian Ocean islands (including Mayotte) CVA24v outbreaks [9,10,12,32,34]. Despite this genetic divergence at the global scale, both clades (i.e., Asia and Africa) drove large-scale, near-synchronous outbreaks. For this reason, viral evolution alone is unlikely to explain the parallel resurgence. Previous immunologic studies have demonstrated that immunity to CVA24v declines rapidly after infection (approximately less than 1 year) [30], leading to replenishment of new susceptible individuals in addition to births. Seasonal and environmental factors may also influence emergence of CVA24v outbreaks [45–47]. For instance, CVA24v-associated AHC incidence in China showed a strong correlation with warm and humid months [47], implying that weather conditions enhance viral stability or transmission via fomites and ocular secretions. Similarly, the Kenyan outbreak occurred during warm, humid months, and a period marked by unusually strong winds. Hence, it is likely that multiple factors converge to spark a new CVA24v epidemic: antigenic novelty, waning of previous immunity and environmental conditions that support virus particle survival and dispersal [12,28].

The viruses sampled in Kenya, Mayotte and Malawi in 2024 were highly similar and closely related to a virus from Uganda (2017). The Ugandan 2017 sequence exhibited the same intra-species recombination signal in the 3D *pol* region that was present in the 2024 Kenya, Malawi and Mayotte sequences, supporting the hypothesis of a recent shared ancestry of CVA24v strains in Eastern/Southern Africa. It is difficult to discern whether these viruses were cryptically transmitting in the region and hence undetected [12,33] or were reseeded from unknown source. The nucleotide similarity of the viruses sampled within Eastern Africa region and the estimated tMRCA suggest that the 2024 CVA24v epidemic strains likely circulated for a few months prior their first detection early in 2024. The continued sporadic detection of positive cases in coastal Kenya long after the peak of the outbreak supports the hypothesis that CVA24v may continue to circulate cryptically during inter-epidemic times albeit at low levels, remaining un-detected.

CVA24v is currently classified into eight genotypes (G-I–VIII), although all recent studies discussed here have only detected genotype G-IV. Within genotype IV, we observed substantial genetic divergence, highlighting that the current classification needs to be updated to better track emerging genotypes. We propose systematic re-evaluation of the current CVA24v genotyping scheme to better describe and reflect its recent genomic epidemiology. Additional new genotypes/ sub-genotypes/lineage should be assigned.

The finding that the recent CVA24v strains from Kenya, Mayotte, Malawi and Uganda likely have a recombinant origin highlights the complex evolutionary history of CVA24v in the Eastern African region. Our expanded comparative analysis identified a possible intra-species recombination event between the Eastern Africa CVA24v and another enterovirus lineage within the 3D^*pol*^ gene. This finding has important implications for our understanding EV-C evolution. Intra-species recombination among EV-Cs has been reported previously [48]. PV2 circulating vaccine-derived poliovirus (cVDPV) strains isolated in Madagascar between 2002 and 2005 exemplify this pattern, exhibiting complex mosaic genomes apparently derived from simultaneous recombination with multiple EV-C species. Sequencing demonstrated that the P2 (2A, 2B and 2C) and 3D^*pol*^ regions of these cVDPV were derived from circulating CVA17 and CVA13 strains, with sustained recombination signals persisting through 2005 involving CVA13, CVA17, and possibly CVA11 [48–50]. Hence, intra-species genetic exchange is an ongoing evolutionary process in EV-C. That the recombination event described here involves sequences from multiple geographic locations (Kenya, Mayotte, Malawi) and time points (2017 Uganda to 2024 Kenya) suggests a single recombination event followed by geographic spread. The mechanistic basis of this recombination event merits further investigation.

Also of note was the evidence for an accumulation of amino acid changes relative to the prototype strain, reflecting continuous evolution of CVA24v since its discovery in 1970 [13,26]. As expected, CVA24v evolution is dominated by strong purifying selection, with only a small number of codon positions showing signature of adaptive change. Indeed, across the three different methodologies (FEL, SLAC, FUBAR) we consistently detected a limited number of codon positions under positive selection, particularly codon sites in VP1 (683, 830, 871, 881), 2C (1371), and 3C (1601). Unfortunately, there is currently limited functional or structural mapping data available for these residues in CVA24v, but their occurrence highlights an important area for future investigation of the structural context and potential epitope associations of these residues.

Our phylodynamic analysis revealed marked temporal fluctuations in CVA24v effective population size (*Ne*) over time. An increase in *Ne* was observed in the early 1970s, corresponding to the emergence and global spread of CVA24v during the first recorded outbreaks of AHC in Singapore, China and Taiwan [17,51]. Subsequent peaks in *Ne* corresponded to documented epidemic waves in the mid-1980s (China and Thailand), early 2000s (Korea India and Philippines), late 2000s (China and Cambodia), and mid-2010s (mainly in Southeast Asia and South America). Each of these expansion phases was followed by sharp declines in *Ne*, consistent with inter-epidemic periods characterized by reduced transmission and limited genomic sampling. The most recent rise in *Ne* during 2023–2024 coincided with widespread CVA24v activity across Eastern/Southern Africa, the Indian Ocean islands, and South-East Asia. Such phylodynamic patterns are consistent with CVA24v’s epidemiological signature of short, explosive outbreaks interspersed with prolonged periods of reduced transmission/non-detection [23,26].

This study has some limitations. First, almost all participants in the study were recruited with a goal of identifying viral acute respiratory infection or diarrheal illness disease agents but not AHC. For instance, symptoms consistent with AHC were not systematically documented and thus clinical epidemiology of identified strains cannot be fully investigated here. Consequently, the more refined CVA24v epidemiological patterns of this infection at the population level might require future focused investigation of CVA24v/AHC. Second, there was limited availability of representative CVA24v genomic sequences from outbreaks in neighboring countries and across the Eastern/Southern Africa region. This paucity of regional genomic data reduces the power to fully resolve transmission routes/pathways, infer viral introductions, or contextualize local viral diversity within broader African continent or globally. Third, the CVA24v qPCR primers used in this study can cross-react with other enteroviruses, such as CVA13 and EV-C99. As a result, some qPCR positives were ultimately found not to represent CVA24v. This highlights the need for confirmatory partial or WGS to confirm CVA24v presence in samples.

In conclusion, by complete or near-complete genome sequencing a large set of CVA24v sequences we demonstrate that the 2024 CVA24v outbreak in coastal Kenya was driven by two distinct CVA24v sequence clusters within genotype G-IV that have experienced sporadic adaptive evolution and a history of recombination. The identified two CVA24v lineages that likely diverged around mid-2023, clustered closely with contemporaneous CVA24v sequences sampled in Eastern/Southern Africa and Indian Ocean Islands yet were genetically distinct from other G-IV clade viruses sampled in Asia in 2023. More broadly, our analysis demonstrates the value of genomic data in revealing epidemic interconnectedness and in designing potential public health control measures.

## Methods

### Ethics statement

Each surveillance platform (community, outpatient, and in-patient) that provided samples analysed here had a dedicated research protocol. The protocols were reviewed and approved by the Kenya Medical Research Institute Scientific Ethics and Research Unit (KEMRI SERU), Nairobi, Kenya (protocol numbers #3178, #3103 and #4724). Written informed consent was obtained from a parent or guardian for participants aged below 18 years. In addition, three ocular samples analysed in this study were collected during the Ministry of Health’s Acute Haemorrhagic Conjunctivitis (AHC) outbreak response, where written informed consent is not required under public health emergency provisions; these samples were fully anonymised, and their use for sample processing was approved by SERU under protocol #KEMRI/SERU/CGMR-C/304/4894 (approved 19 May 2024). All the experiments in this study were performed in accordance with relevant guidelines and regulations.

### Study design and population

The samples analysed in this study were collected through multiple surveillance platforms on the Kenyan Coast run by KEMRI-WTRP and are of varied study design. These included disease outbreak response activities [32] and ongoing acute respiratory/diarrhoea disease surveillance within the KHDSS [39] (Fig A in S1 Appendix). The details of the contributing studies are provided below:

*ResViRe (Respiratory Virus Reinfections) study*: A community-based homestead surveillance study. This was initiated in August 2023 and was ongoing throughout 2024 across five administrative locations: Tezo, Roka, Zowerani, Matsangoni, and Ngerenya. Recruited homestead participants were visited weekly at home and their respiratory disease status and travel history recorded, with nasopharyngeal and oropharyngeal (NP/OP) swabs taken regardless of symptom status. Whenever one or more homestead member(s) were positive for severe acute respiratory syndrome coronavirus 2 (SARS-CoV-2), respiratory syncytial virus (RSV) or influenza A/B, sample collection frequency for the homestead was increased to twice a week until all members test negative. By December 31, 2024, 67 homesteads (713 participants) had been enrolled and sampled at least once.

*Paediatric pneumonia in-patient (IP*) surveillance study at Kilifi County Referral Hospital (KCRH). This has been ongoing since 2002 [52]. Respiratory samples are collected from children aged <5-year-old admitted to KCRH with clinical symptoms of severe or very severe pneumonia as per WHO guidelines [53].

*Health facility out-patient (OP) acute respiratory infection (ARI) surveillance study.* This began in December 2020 and was ongoing throughout 2024 [54]. The study was undertaken at five selected health facilities with the KHDSS: Matsangoni, Mavueni, Pingilikani, Mtondia and the KCRH out-patient department. At each health facility up to 15 NP/OP swabs are collected each week from persons of all ages presenting with ARI [55].

*The IP childhood diarrhoea aetiology (enteric pathogens) surveillance study.* This began at KCH in September 2009 and was ongoing throughout 2024. The study was established to track rotavirus infections/disease burden in the KHDSS population to provide impact data following introduction of rotavirus vaccination programme. Stool samples are collected from all eligible children <13 years of age admitted to the paediatric ward with diarrhoea as one of their presenting symptoms.

### CVA24v detection and partial sequencing

CVA24v was identified in clinical samples using a qPCR and/or sequencing of the VP4/VP2 genomic region of the enterovirus genome. Samples collected through ResViRe study between August 2023 and June 2024 were initially screened for rhinovirus using a qPCR assay. This assay targets the 5′ untranslated region (5′UTR) using the following primers and probes; forward primer (TGGACAGGGTGTGAAGAGC, reverse primer (CAAAGTAGTCGGTCCCATCC) and probe (VIC-TCCTCCGGCCCCTGAATG-TAMRA) and cross-reacts with range of enteroviruses including CVA24v. A negative control (NC) (nuclease-free water) and positive control were included in the assay. Samples were considered positive if amplified with Ct value < 35.0. VP4/VP2 genomic region in the positive samples were subsequently amplified using forward primer (CCGGCCCCTGAATGYGGCTAA) and reverse primer (TCWGGHARYTTCCAMCACCANCC) and sequenced on the Illumina MiSeq platform. Raw Illumina reads were quality-filtered (Phred score < 20) and adapter-trimmed using fastp, followed by *de novo* assembly with SPAdes. The contigs were mapped to a reference HRV genome before generation of consensus sequences*.* Resultant VP4/VP2 sequences were then analysed using the Enterovirus Genotyping Tool [36] to assess the presence of CVA24v in the samples. Samples collected after June 2024, as well as those from other studies, were retrospectively screened using a CVA24v specific qPCR assay adapted from Lévêque et al [56]. The assay targets the 5′UTR and uses the following primers: forward primer 5′-CCAACCACGGAGCAGGTGA-3′, reverse primer 5′-GAAACACGGACACCCAAAGTAGT-3′, and probe 5′-CAACCCAGCAACTAGCCTGTCGTAACGC-3′.

### Whole-genome sequencing of CVA24v

Whole-genome sequencing from CVA24v-positive samples was performed using a three amplicon-targeted sequencing protocol developed in our laboratory [33]. Briefly, viral RNA was reverse transcribed using the LunaScript RT SuperMix Kit (New England Biolabs). A negative control (NC) (nuclease-free water) was included during both the extraction and reverse transcription steps. The cDNA was then amplified in three reaction tubes with the Q5 Hot Start High-Fidelity 2Master Mix (NEB) with optimised CVA24v primers and thermocycling conditions as previously described [33]. The PCR products were loaded on a 1.5% agarose gel to confirm amplification before purification using Agencourt AMPure XP beads. Library preparation was performed using the Ligation Sequencing Kit (SQK-LSK114) and Native Barcoding Kit (NBD96), and sequencing performed on the Oxford Nanopore Technologies (ONT) GridION platform. The reduction in the number of PCR-positive samples to successfully sequenced whole genomes was primarily due the generic quality constraints (e.g., poor RNA quality) and cross-reactivity of the qPCR assay with other enteroviruses, such as CVA13 and EVC99. To increase genome recovery yields, strict priority was given to samples with qPCR cycle threshold (Ct) value <30.0.

### Genome assembly

Genome assembly was performed using a sub-workflow of an in-house bioinformatics pipeline named *ViralPhyl*v0.11.2 and available on GitHub (https://github.com/kwtrp-peo/viralphyl). Base-called reads were demultiplexed using the ARTIC Guppyplex tool with default parameters, applying a minimum Q score of 9. Reads shorter than 400 nt were filtered out using the toullingQC module. Consensus sequences were generated by aligning the reads to a reference sequence (in this case CVA24_2400060741_FRA24, GenBank accession PP548240). The reads were aligned using MiniMap2 [57]. Positions with genome coverage below 20 reads were masked with ambiguous nucleotide (‘N’). The resulting consensus sequences were further refined using Clair v 2.1.1 [58].

### Global comparison data set

All CVA24v sequence available in GenBank (https://www.ncbi.nlm.nih.gov/genbank/) as of November 01, 2025, and with >90% coverage were retrieved. This data set was used to assess the genetic relatedness of Kenyan CVA24v to global strains, identify potential geographic origins, and infer patterns of lineage emergence, persistence, or reintroduction in Eastern Africa. In total,159 genomes (>6500 bp) sampled between 1970 and 2024 from 21 countries across Africa, Asia, the Americas, and island nations in the Pacific Oceans were included in the analysis (Fig 2E). Additionally, 418 available partial VP1 sequences were downloaded and included specifically for assessing clustering patterns and classification among CVA24v genotype IV (G-IV) sequences.

### Sequence alignment and variation analysis

All the retrieved global CVA24v sequences were collated and aligned using MAFFT v7.520117 [59]. Nucleotide (nt) and amino acid (aa) variations/homology/similarity were subsequently calculated using MEGA v11.0 [60] and the dist.dna() function in the ape5.0 [61] in R. To identify specific amino acid changes, the Kenyan sequences were compared against the prototype strain EH24/70 (GenBank accession no. D90457). The nt and aa variations were analysed and visualised in Snipit v1.618 [62]. For clustering using partial VP1 sequences, sequences were clustered at 95% nucleotide identity threshold (i.e., 0.05 divergence). This 0.05 threshold was chosen because it provided well defined clusters consistently supported by high bootstrap values (>70%)

### Phylogenetic analysis

To determine the global phylogenetic structure and evolutionary dynamics of CVA24v, the Kenyan CVA24v sequences were analyzed together with the 159 publicly available genomes (>90% coverage) retrieved from GenBank (as of 01 November 2025). To avoid over-representation of closely related sequences and optimally capture viral diversity, the Kenyan genomes (n = 245) were sub-sampled to account for repeated sampling from the same individual and homestead where samples were likely to be epidemiologically linked. In such cases, a single representative genome, typically the earliest sampled or highest-quality sequence, was retained, resulting in 65 sequences for downstream phylogenetic and phylodynamic analyses. Sequence alignment was performed using MAFFT v7.520 [59] with the --auto strategy used to optimize alignment parameters for nucleotide data. The aligned data set was manually inspected and trimmed in AliView v1.30 [63]. A maximum likelihood (ML) phylogenetic tree of this alignment was then inferred using IQ-TREE v2.1.3 (http://www.iqtree.org/) under the General Time Reversible (GTR + Γ) nucleotide substitution model, as determined by ModelFinder [64]. Node support was evaluated using 1,000 ultrafast bootstrap replicates, and the resulting tree was visualised and annotated using FigTree v1.4.4 [65] and ggtree v3.16.3 [66] in R [67].

### Phylodynamic analysis

The extent of temporal signal in the sequence data was first assessed using TempEst v1.5.3 [68] by performing a regression of root-to-tip genetic distances against sampling dates. Estimation of the nucleotide substitution (evolutionary) rate and Time to the Most Recent Common Ancestor (tMRCA) was then performed using the Bayesian Markov Chain Monte Carlo (MCMC) approach available in the Bayesian Evolutionary Analysis Sampling Tree (BEAST) package (v1.10.4 31) [69] as well as in TreeTime [70]. Bayesian MCMC analyses were run for 500 million generations, with sampling performed every 1000 generations. Convergence of parameters was assessed in Tracer v1.7.1 (http://tree.bio.ed.ac.uk/software/tracer/, and was considered to have been achieved when the effective sample size (ESS) of key parameters > 200. In all cases, the initial 10% of the run was used as “burn-in”. A Maximum Clade Credibility (MCC) tree was generated using TreeAnnotator v1.8.4, and the resulting phylogeny visualized in FigTree v1.4.4 [65] or ggtree v3.16.3 [66] in R [67]. To infer temporal changes in viral genetic diversity through time values of the effective population size (*Ne*) were estimated using a skyline coalescent model [70].

### Natural selection analysis

Selection pressures across the coding region of all CVA24v sequences, including Kenyan strains, were assessed using HyPhy 2.5 [71], applying multiple detection methods: FEL, SLAC, and FUBAR. FEL was used to identify codons under pervasive positive (d_N_ > d_S_) and negative selection (d_N_ < d_S_). SLAC provided estimates of d_S_ and d_N_ across the alignment based on maximum likelihood ancestral reconstruction, while FUBAR estimated codon-specific differences between nonsynonymous and synonymous rates under a Bayesian framework. For SLAC and FEL, significance was defined as p < 0.1, whereas for FUBAR, a posterior probability ≥ 0.9 indicated evidence of selection.

### Recombination analysis

Potential recombination events were investigated using a combination of the (Recombination Detection Program) RDP5 [72] and SimPlot++ [73]. To ensure robust characterisation of potential intra-species recombination events, we conducted analyses using representative a 2024 Kenyan CVA24v sequence (GenBank accession number: PX659173) as a query sequence against a global data set of 2418 complete or near complete EV-C genomes. This expanded data set included poliovirus types 1, 2, and 3 (PV1–3) along with a broad representation of non-polio EV-C reference sequences. Aligned genomes were screened with multiple algorithms in RDP5, namely RDP, GENECONV, BootScan, MaxChi, Chimaera, SiScan, and 3Seq, applying Bonferroni-corrected p-values < 0.05 to confirm recombination signals detected by at least three methods. Breakpoints were mapped relative to the CVA24v reference genome, and parental lineages were inferred where possible. Recombination breakpoints were visualised in SimPlot analyses with a 200 bp sliding window and a 20 bp step size. To confirm putative recombination events with other *Enterovirus C* strains, phylogenetic trees were estimated for regions on either side of the breakpoints identified to assess topological incongruence.

## Supporting information

S1 Appendix Appendix file contains Table A and Fig A-E. (DOCX)
