# Supplementary material for: Genomic epidemiology of coxsackievirus A24 variant during the 2024 acute hemorrhagic conjunctivitis outbreak in Coastal Kenya
Source: PLoS Pathog. 2026 Sep 15;22(9):e1014589. doi: 10.1371/journal.ppat.1014589 (PMC13592715; doi:10.1371/journal.ppat.1014589)
Supplement: Supplementary file 1 [file ppat.1014589.s002.pdf]

Review by Emma Hodcroft

In this paper, "Genomic Epidemiology of Coxsackievirus A24 Variant During the 2024 Acute Hemorrhagic Conjunctivitis Outbreak in Coastal Kenya", the authors undertake an impressive diagnostic, sequencing, and analysis effort to contribute to a better understanding of the circulation and diversity of CV-A24, particularly in an African setting. Overall, I found this paper well-written and thorough, and a valuable contribution to our understanding of one of many under-studied enteroviruses. In particular, it's fantastic to see the increase in number of available A24 sequences, and worth noting that the authors have already made these sequences publicly available. The majority of the analyses are appropriate and informative. Most of my comments are minor, though I have one substantive concern with the recombination analysis that I believe requires revisiting in full before publication.

Recombination:

My largest issue with the manuscript is with the recombination analysis. I am not yet convinced that it is clearly shown that there is a potential recombination with poliovirus 1. I believe the analysis needs to be repeated using one of the putative recombinant sequences as the query sequence, including different poliovirus sequences, and including more non-polio EV-C sequences.

In Figure 6 A, it is unclear what the query sequences is (the similarity against which the other sequences are plotted). I would have expected it to be potentially one of the Kenya 2024 sequences, but Kenya_2024 is instead listed as one of the references. When the legend refers to 'Kenya_2024' is this one example sequence or a consensus – if the latter how was it generated? (Similar questions for the other sequences included.) What is the cluster of the references included (e.g. C1/1/24, C1/2/24, C1/3/24)? How were the particular polio sequences included chosen?

Figure 6 A shows a drop in similarity between the query (as well as other A25v viruses) and Kenya_2024, Uganda_2017, Malawi_2024, and Mayotte_2024, at just after 5100bp, which could indicate recombination. These sequences drop to the same level of similarity as the included poliovirus sequences. Importantly this does not necessarily indicate that the Kenya/Uganda/Malawi/Mayotte sequences are similar to the polio sequences – it rather indicates that they and the polio sequences are roughly equally divergent from the query. This plot should really be inverted – traditionally one would make the query the suspected recombinant sequence, meaning similarity is then relative to the suspected recombinant. This is important as the graph shows distance from the query sequence, not similarity to the other references – 2 sequences could both be 20% distant from the query, but not be identical to each other (indeed, they

could be very diverged). Thus, to show that the putative recombinant sequences are very similar to the poliovirus sequences, one needs to invert the plotting.

Equally importantly, no other non-polio EV-Cs are included in the graph. This is critical, as it's impossible to determine if the distance between these four sequences/sequence groups (Kenya/Uganda/Malawi/Mayotte) and the polio sequences is close, or just closer relative to the query (perhaps any other EV-C sequence would be equally 'distant' from the query if others were plotted?). After inverting the graph, including other non-polio EV-Cs will help to clarify if there are any closer putative parents, or indeed if other EV-C sequences are as close (or distant) from the query as the polio sequences.

Notably the Malawi_2024 sequence has a sharp increase in similarity compared to the others (Kenya/Uganda/Mayotte) shortly after the drop in similarity – why is this? Also, there's a small gap visible in the graph just before this – what is this?

In such plots, it's also important to remember that EVs are relatively similar (particularly within type), and that similarity varies greatly across the genome – with some areas being more conserved than others. Finding diversity of 70-80% (line 260) between EV-Cs is not necessarily unusual, particularly in this region.

The authors then move directly to an RDP5 analysis, but again seemingly only include polio sequences (and only poliovirus 1 is shown in Fig 6 B) in their analysis. (Please label all potential parents directly in Fig 6 B – it's not clear to me why there are 3 lines.) This means it is not possible to conclude that another EV-C, or indeed that PV-2 or 3, could not be the recombination partner. The same is true for the phylogenetic trees made with sections of the genome (Figure 7), which only include poliovirus sequences. (In this figure it is also impossible to see the PV-1 sequence(s?) and thus difficult to assess whether PV-1 may group more closely than PV-2 or PV-3).

Beyond the need to include more EV-C sequences, there is a further complication that is worth considering when interpreting the polio sequences already included in the analysis. It's important to remember that poliovirus sequences themselves are often recombinants. In particular, for the polio sequences used in Fig 6 A), our independent pre-existing analysis of EV-C genomes shows there's a good chance these sequences are recombinant themselves in the 3D gene. This graph (from existing internal work) shows a tanglegram of VP1 vs 3D, coloring sequences by their VP1 type, and shows that in the 3D gene, many poliovirus 1, 2, and 3 sequences split in their associations between VP1 and 3D.

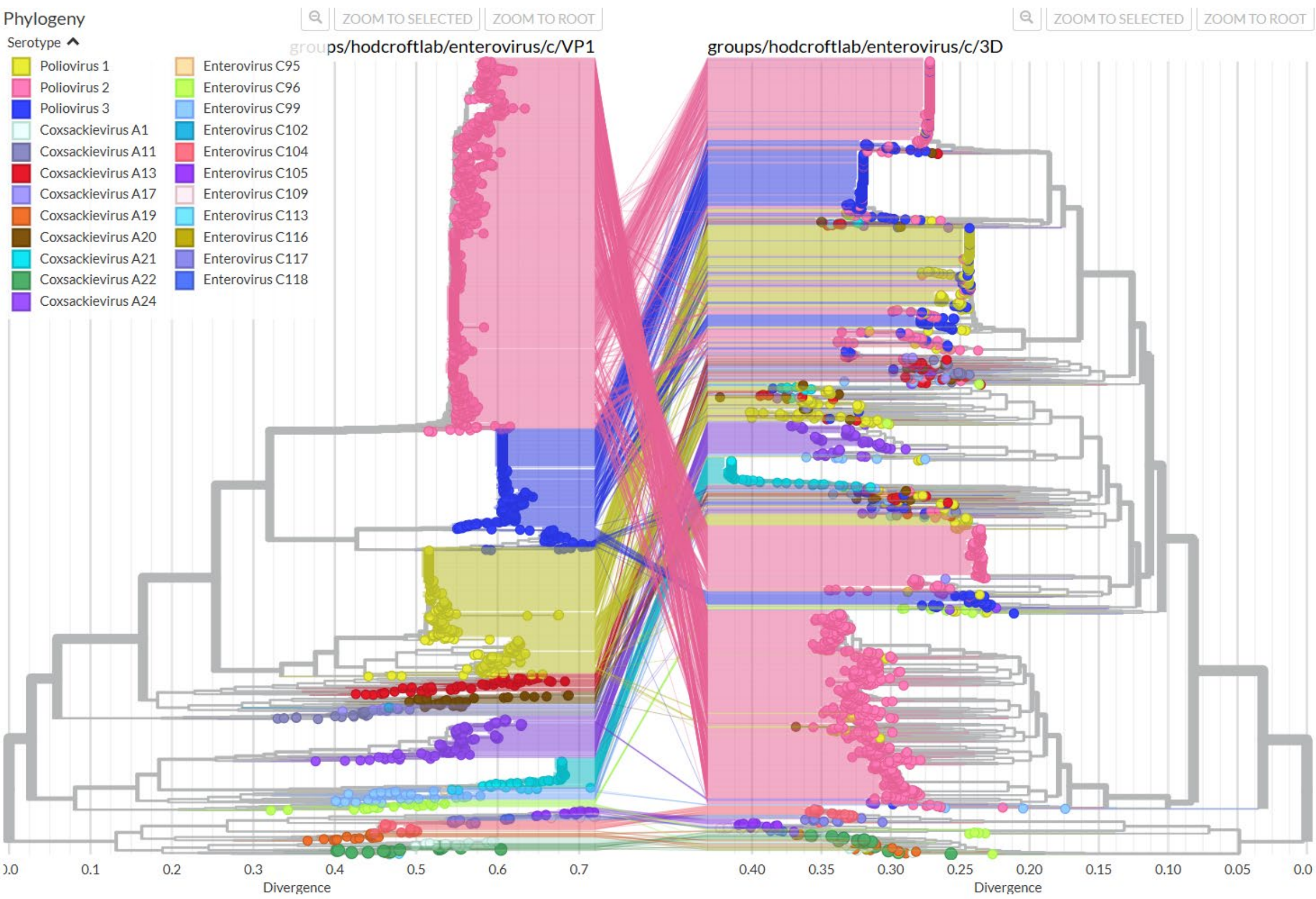

Highlighting the specific sequences used in Fig 6 A) (highlighted in blue) it's possible to see that the PV1 and PV3 sequences follow the minority of the clade and split to the lower part of the tree, close to part of PV2 (in the case of PV1, actually interleaving with PV2).

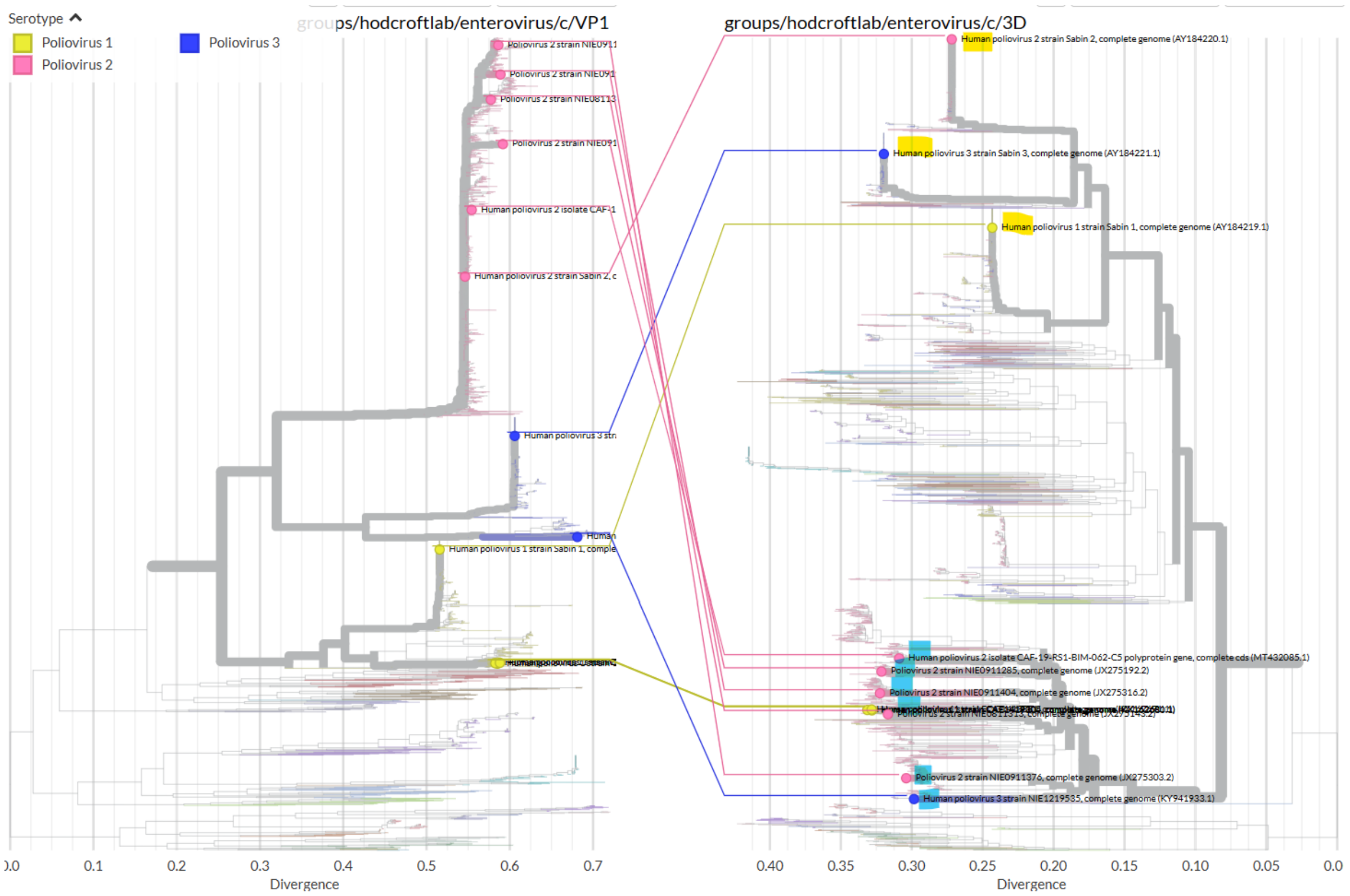

Further, if we assume the A24v recombination partner is most likely derived from a vaccine-derived polio virus (originally from the Sabin vaccines), since endemic wild polio was last detected in 1984, we would expect the 3D segment of A24v to be closer to the Sabin genome. However, the Sabin genomes (highlighted in yellow) are instead quite distant from the included poliovirus sequences that are 'similar' to the A24v sequences. Thus it seems very possible that all of the included poliovirus sequence may be recombinant themselves. The recombination partner for these poliovirus sequences and the A25v sequences could then perhaps be an unknown EV-C (or multiple such viruses, relatively closely related) and have recombined with these viruses separately. Alternatively, perhaps the original recombination occurred in PV2, and this recombinant has then recombined again with A24v (but importantly, it doesn't seem to be the original Sabin2 3D which is the recombination partner).

Additionally, Figure 6 A is quite difficult to read – the colours are not distinguishable and the lines often obscure each other. I would suggest showing only the most vital and convincing sequences or sequence-groups (perhaps 10) on the main-text figure and then including more plots as supplemental figures, showing similarity to other sequences or groups of sequences. And in line with my suggestion above, it would be good to include other non-polio EV-C viruses in supplementary figs (e.g. Fig S4) – and ensure PV1 sequences are visible (and potentially include more of them?).

Finally, on a related note, if such a recombination is present, this will also influence the estimate of the MRCA for any whole-genome datasets that include both recombinant and non-recombinant sequences (estimating the MRCA for only recombinant sequences should be ok). Thus for the global MRCA estimates (section starting line 187) the authors should consider masking the recombinant region or consider alternative ways to address this.

Smaller issues:

- Given the confusing naming of Enteroviruses in general, it would be worth clarifying in the introduction that A24 is an EV-C (the name unfortunately could convey it is an EV-A)
- CV-A24v is a variant or subclade of CV-A24, but this relationship isn't made very clear in the paper ("caused by Coxsackievirus A24 variant" could be read as just being the A24 variant of the larger EV-C group), and would be worth being a little more explicit about. It would be useful to hear what proportion of A24 is A24v and if there's any general information about how the subclade is defined (e.g. by specific mutations).
- In the results (line 161) it's mentioned there are 8 identified genotypes of A24v– it would be helpful to mention this earlier, in the introduction.

- In Table 1, it was a little hard to match up the numbers with those mentioned in the text. For example, WG sequencing was attempted on 333 samples (line 148), presumably out of 178+602 (Table 1) positives. Was the reduced number primarily because of the CT threshold mentioned, or due to false-positives meaning some of the positive samples were actually other EVs (cross-reactivity, mentioned on line 386-388), or other reasons?
- In line 111-2 it's mentioned that sequenced samples were combined with all publicly A24v available data – is this all publicly available whole-genomes, or any length? How did the authors identify public sequences as being A24v rather than A24?
- If possible it would be interesting to dig a little more into the amino-acids under selection (section starting line 222) – are any of these surface exposed, or suspected/confirmed epitopes? Is anything else known about these locations?

Minor comments/typos:

- Line 90 – 'viral capsid (VP) protein' should probably be 'viral capsid protein (VP)'
- Line 107-8 – It would be good to reference Fig S5 here so that readers can orient themselves with the geography
- Line 112 – Specify if only WGS publicly available genomes were included (or also partial genomes)
- Line 113 – 'which largely focused descriptive' → 'which largely focused **on** descriptive'
- Line 125 – 'peak CV-A24v was observed' – perhaps 'samples' or 'cases' is missing?
- Line 137 – 'were more frequently in infants' → 'were more **frequent** in infants'
- Line 138-140 – Is it possible to add these locations to the map in Fig S5?
- Line 168 – 'revealed viruses' → 'revealed **that** viruses'
- Line 172 – Does 'dereplicated' mean 'deduplicated'?
- Line 182 – 'they close to the' → 'they **are** close to the'
- Line 366 – Closing parenthesis missing after 'South America'
- Figure 1 – Please add labels to the axes and indicate more clearly which corresponds to the line & which to the bars. In addition, the red and green are sometimes hard to distinguish on some screens – and very difficult to distinguish for red/green colour blind
- Figure 2, the slope is given as 5.17e-03, but in the text the rate is given as 5.37x10^-3 (line 191) – possible typo?
- Figure 2 E – The colours used in the world map are a little contrary to norms – usually yellow/red are 'hotter' and therefore 'more' and blue represents 'less'
- Figure 3 – Please add labels to the axes
- Figure 3 B – The text says there are 22 distinct sub-groups (line 174), but only 17 are shown in the figure. Among the missing are C12, C14, C15, C21

- Figure S1 B – the labels are cutoff on the right, one can’t see the last letter of the clustering groups
