# Supplementary material for: Genomic epidemiology of coxsackievirus A24 variant during the 2024 acute hemorrhagic conjunctivitis outbreak in Coastal Kenya
Source: PLoS Pathog. 2026 Sep 15;22(9):e1014589. doi: 10.1371/journal.ppat.1014589 (PMC13592715; doi:10.1371/journal.ppat.1014589)
Supplement: Supplementary file 2 [file ppat.1014589.s001.docx]

Supplementary Information for

Genomic Epidemiology of Coxsackievirus A24 Variant During the 2024 Acute Hemorrhagic Conjunctivitis Outbreak in Coastal Kenya

John Mwita Morobe^1,2 *^, Edidah M. Ong'era^1^, Arnold W. Lambisia^1,2^, Samuel O. Odoyo^1^, Martin Mutunga^1^, Esther N. Katama^1^, Robinson Cheruiyot^1^, Joyce U. Nyiro^1^, Everlyn Kamau^3^, Charlotte J. Houldcroft^4^, Matt Keeling^5^, Katherine Gallagher^1,6^, Edward C. Holmes^7^ & Charles N. Agoti^1,8^

^1^Kenya Medical Research Institute (KEMRI)-Wellcome Trust Research Programme, Kilifi, Kenya.

^2^The Open University, Milton Keynes, United Kingdom.

^3^University of California San Francisco, United States of America.

^4^Department of Genetics, University of Cambridge, Cambridge, United Kingdom.

^5^The Zeeman Institute for Systems Biology and Infectious Disease Epidemiology

Research (SBIDER), University of Warwick, Coventry, United Kingdom.

^6^Department of Infectious Diseases Epidemiology, London School of Hygiene and Tropical Medicine, London, United Kingdom.

^7^School of Medical Sciences, The University of Sydney, Sydney, New South Wales, Australia.

^8^School of Public Health, Pwani University, Kilifi, Kenya.

**This file includes:**

S1 Appendix Table A

S1 Appendix Figs A-E

**Table A**. Demographic and baseline characteristics of participants across different surveillance platforms

|  |  | **ResViRe** | |  |  | |  |  |
| --- | --- | --- | --- | --- | --- | --- | --- | --- |
| **Characteristic** | **Overall N = 714** | **CVA24v negative N = 496** | | **CVA24v positive N = 218** | **p-value** | |  |  |
| Sex |  |  | |  | 0.3 | |  |  |
| Female | 408 (57%) | 290 (58%) | | 118 (54%) |  | |  |  |
| Male | 306 (43%) | 206 (42%) | | 100 (46%) |  | |  |  |
| Median Age (years) | 14 (4, 29) | 19 (8, 35) | | 5 (1, 12) | <0.001 | |  |  |
| Age Category (Years) |  |  | |  | <0.001 | |  |  |
| 0-5 | 207 (29%) | 95 (19%) | | 112 (51%) |  | |  |  |
| 6-10 | 97 (14%) | 55 (11%) | | 42 (19%) |  | |  |  |
| 11-20 | 143 (20%) | 115 (23%) | | 28 (13%) |  | |  |  |
| 21-30 | 94 (13%) | 78 (16%) | | 16 (7.3%) |  | |  |  |
| 31-40 | 77 (11%) | 68 (14%) | | 9 (4.1%) |  | |  |  |
| 41-50 | 34 (4.8%) | 32 (6.5%) | | 2 (0.9%) |  | |  |  |
| >50 | 62 (8.7%) | 53 (11%) | | 9 (4.1%) |  | |  |  |
| Location |  |  | |  | 0.015 | |  |  |
| Matsangoni | 132 (18%) | 93 (19%) | | 39 (18%) |  | |  |  |
| Ngerenya | 144 (20%) | 103 (21%) | | 41 (19%) |  | |  |  |
| Roka | 141 (20%) | 87 (18%) | | 54 (25%) |  | |  |  |
| Tezo | 151 (21%) | 119 (24%) | | 32 (15%) |  | |  |  |
| Zowerani | 146 (20%) | 94 (19%) | | 52 (24%) |  | |  |  |
|  |  |  | |  |  | |  |  |
| **Health facility out-patient surveillance** | | | | | | |  |  |
| **Characteristic** | **Overall N = 1,486** | **CVA24v negative N = 1,420** | | **CVA24v positive  N = 66** | **p-value** | |  |  |
| Sex |  |  | |  | 0.087 | |  |  |
| Female | 871 (59%) | 839 (59%) | | 32 (48%) |  | |  |  |
| Male | 615 (41%) | 581 (41%) | | 34 (52%) |  | |  |  |
| Median Age (years) | 16 (4, 30) | 16 (4, 31) | | 9 (2, 18) | 0.004 | |  |  |
| Age Category (Years) |  |  | |  |  | |  |  |
| 0-5 | 444 (30%) | 414 (29%) | | 30 (45%) |  | |  |  |
| 6-10 | 155 (10%) | 150 (11%) | | 5 (7.6%) |  | |  |  |
| 11-20 | 315 (21%) | 300 (21%) | | 15 (23%) |  | |  |  |
| 21-30 | 203 (14%) | 195 (14%) | | 8 (12%) |  | |  |  |
| 31-40 | 130 (8.7%) | 127 (8.9%) | | 3 (4.5%) |  | |  |  |
| 41-50 | 81 (5.5%) | 79 (5.6%) | | 2 (3.0%) |  | |  |  |
| >50 | 158 (11%) | 155 (11%) | | 3 (4.5%) |  | |  |  |
| Facility |  |  | |  | 0.042 | |  |  |
| Kilifi County Hospital | 448 (30%) | 427 (30%) | | 21 (32%) |  | |  |  |
| Matsangoni | 185 (12%) | 181 (13%) | | 4 (6.1%) |  | |  |  |
| Mavueni | 312 (21%) | 296 (21%) | | 16 (24%) |  | |  |  |
| Mtondia | 199 (13%) | 196 (14%) | | 3 (4.5%) |  | |  |  |
| Pingilikani | 342 (23%) | 320 (23%) | | 22 (33%) |  | |  |  |
|  |  |  | |  |  | |  |  |
|  |  |  | |  |  | |  |  |
| **Pediatric in-patient pneumonia surveillance** | | | | | | |  |  |
| **Characteristic** | **Overall N = 391** | **CVA24v Negative N = 327** | | **CVA24v Positive  N = 64** | **p-value** | |  |  |
| Sex |  |  | |  | 0.12 | |  |  |
| Female | 179 (46%) | 144 (44%) | | 35 (55%) |  | |  |  |
| Male | 212 (54%) | 183 (56%) | | 29 (45%) |  | |  |  |
| Median Age (Months) | 11 (8, 17) | 11 (8, 17) | | 11 (7, 21) | >0.9 | |  |  |
| Age Category (Months) |  |  | |  | 0.015 | |  |  |
| 0–5 | 52 (13%) | 40 (12%) | | 12 (19%) |  | |  |  |
| 6–11 | 147 (38%) | 124 (38%) | | 23 (36%) |  | |  |  |
| 12–23 | 138 (35%) | 124 (38%) | | 14 (22%) |  | |  |  |
| 24–59 | 38 (9.7%) | 26 (8.0%) | | 12 (19%) |  | |  |  |
| >60 | 16 (4.1%) | 13 (4.0%) | | 3 (4.7%) |  | |  |  |
|  |  |  | |  |  | |  |  |
|  |  |  | |  |  | |  |  |
| **In-patient diarrhea etiology surveillance** | | | | | | |  |  |
| **Characteristic** | **Overall  N = 1,088** | **CVA24v negative N = 1,040** | | **CVA24v positive N = 48** | **p-value** | |  |  |
| Sex |  |  | |  | 0.3 | |  |  |
| Female | 477 (44%) | 454 (44%) | | 23 (52%) |  | |  |  |
| Male | 604 (56%) | 583 (56%) | | 21 (48%) |  | |  |  |
| Unknown | 7 | 3 | | 4 |  | |  |  |
| Median Age (Months) | 6 (1, 12) | 5 (0, 12) | | 9 (4, 19) | 0.007 | |  |  |
| Unknown | 7 | 3 | | 4 |  | |  |  |
| Age Category (Months) |  |  | |  | 0.5 | |  |  |
| 0–5 | 538 (50%) | 521 (50%) | | 17 (39%) |  | |  |  |
| 6–11 | 244 (23%) | 233 (22%) | | 11 (25%) |  | |  |  |
| 12–23 | 173 (16%) | 164 (16%) | | 9 (20%) |  | |  |  |
| 24–59 | 126 (12%) | 119 (11%) | | 7 (16%) |  | |  |  |
| Unknown | 7 | 3 | | 4 |  | |  |  |

**Fig A. Study area showing geographic positions of the four surveillance platforms.** Map of Kenya highlighting Kilifi County and the Kilifi Health and Demography Surveillance System (KHDSS). The administrative area of the community household study is shown in light green, while the pink and blue dots indicate the distribution of recruited homesteads in the community household study. Locations of the five health facilities conducting in-patient (KCH) and/or out-patient surveillance are also shown. The map was generated using ArcGIS Desktop 10.8.2 (Esri, Redlands, CA, USA). The base layer (administrative boundary shapefiles for Kenya) was obtained from the Humanitarian Data Exchange (HDX) platform: https://data.humdata.org/dataset/cod-ab-ken. These data are provided by the Kenya National Bureau of Statistics (KNBS): <https://www.knbs.or.ke/portals/>. The associated data policy for KNBS is available at: https://www.knbs.or.ke/wp-content/uploads/2024/07/KNBS-Data-Revision-Policy.pdf, which permits public and academic use. ArcGIS software was used solely as a tool for map generation (https://www.esri.com/en-us/legal/terms/web-site-service) and does not affect the licensing of the underlying spatial data.

**Fig B.** **Time-scaled phylogenetic tree showing the evolutionary placement of Kenyan CVA24v whole-genome sequences within the global diversity of CVA24v.** **(A)** Global CVA24v phylogeny. **(B)** Magnification of the clade containing the Kenyan, Malawi and Mayotte sequences from the global tree. Tip points are colored according to the country of collection. Major clades within the East African region are labelled C1/1/24, C1/2/24 and C1/3/23 to highlight their phylogenetic grouping/clustering.

**Fig C.** **ML phylogenetic trees of CVA24v whole genome sequences sampled during the coastal Kenya the 2024 AHC outbreak (n=245).** (**A**) Tip labels are colored by sampling administrative location within Kilifi county. (**B**) Tip labels are colored by surveillance platforms.

**Fig D**. Amino acid variability in CVA24v whole genome sequences from the Kenyan 2024 and 2010 outbreaks.

**Fig E.** ML phylogenetic trees of different coding regions of CVA24v genome and suspected recombinant viruses. Tip labels colors represent different EV-C species.
