# Supplementary material for: Genomic epidemiology of coxsackievirus A24 variant during the 2024 acute hemorrhagic conjunctivitis outbreak in Coastal Kenya
Source: PLoS Pathog. 2026 Sep 15;22(9):e1014589. doi: 10.1371/journal.ppat.1014589 (PMC13592715; doi:10.1371/journal.ppat.1014589)
Supplement: Supplementary file 3 [file ppat.1014589.s004.docx]

I thank the authors for their attentive incorporation to the reviewer’s suggestions, thoughtful adjustments to the manuscript, and detailed response to the points raised. I find the newly submitted version of the manuscript overall much clearer, particularly with regard to a few of the tables, figures, and clarifications in which data was used where. Overall I am quite satisfied with the review, with one remaining major concern.

Generally I am happy that the authors took the extra time to re-run the recombination analyses as suggested, and believe the new figures and raw results are much more clear than previously. However, I am afraid I remain unconvinced that the recombination partner is Sabin-derived poliovirus type 2 (PV2). As shown in Figure 6A, PV2 does not seem to be any more similar to the query sequence (one of the CVA24v sequences) than the other PVs (or indeed, the presumably non-recombinant CVA24vs). This is further shown in Figure 6B, where while PV2 seems to have *some* identity with CVA24v PX659173, it is considerably lower than the other CVA24v which is the major parent. One drawback of RDP5 analysis, as I understand it, is that it will usually assign parentage from the available parents provided, and rarely calls an ‘orphan state,’ or ‘unknown parent’. This is a real potential drawback as it may obscure any case where a parent cannot be reliably identified.

For a project on EV-Cs (unrelated to this CVA24 work), my group is routinely generating EV-C trees and recombination analyses for most EV-Cs, which I drew upon during my previous review, and will show again here. These include all publicly available, whole-genome EV-C sequences that are not indicated as being patent, lab construct etc.

In short, when plotting similarity, our similarity-plot results for the CVA24v Kenyan outbreak sequences (here the query) agree visually with what the authors show in 6A – that in 3D, the CVA24v Kenyan sequence is recombinant, as its similarity departs from the older CVA24(v) sequences, and that PV1, PV2, and PV3 (Sabin) are all roughly equidistant from the query.

Since the CVA24v sequences are now online, they’ve have been incorporated into our updating phylogenies, and we can now look directly at them in a VP1-3D tanglegram. Below, I’ve filtered to show the 3 Sabin strains (PV1 AY184219; PV2 AY184220; PV3 AY184221), the query sequence from Figure 6B (PX659173), and the two parents from Figures 6B (CVA24v – PX548511; “PV2” - MT432121). (This can also be viewed interactively [here](https://nextstrain.org/groups/hodcroftlab/enterovirus/c/VP1:groups/hodcroftlab/enterovirus/c/3D?s=AY184219.1,AY184220.1,MT432121.1,PX548511.1,PX659173.1,AY184221.1&tl=__strain__))

As is seen, the issue is that MT432121 (“Parent PV2”) is not a Sabin-like strain of PV2 – it itself is recombinant (as can be seen by its placement in 3D far from the Sabin PV2 strain AY184220). Thus, the statement “the 2024 Kenya sequences serving as the major parent and Sabin PV2 as a potential minor parent” is therefore *not* correct – the Sabin PV2 strain is not the donor of the 3D region of PX659173 (Query), and they are very far from each other on the phylogeny. I do not believe there is any evidence that backs up the claim that Sabin PV2 is the recombination parent.

However, as is also visible, even the recombinant PV2 sample MT432121 (“Parent PV2”) is fairly far from the query sequence PX659173. While it’s closer than Sabin PV2, I personally do not believe it is close enough to confidently say it is the recombination partner either, from the phylogeny or from Figure 6B. As I mentioned in my earlier review, I believe it’s likely that the recombination partner for the Kenyan CVA24v samples is likely an unsampled EV-C strain (potentially also the case for the recombinant PV2 sample, MT432121). This can be observed on the tanglegram to some degree, but also in the [3D phylogeny on its own](https://nextstrain.org/groups/hodcroftlab/enterovirus/c/3D?tl=__strain__), where it’s clear that the large number of recombinant PV2 sequences, recombinant PV1 sequences, and CVA24v sequences have no clear association with any other EV-C species.

In short, I do not believe that the recombination conclusion presented - that one of the recombinant parents of the CVA24v Kenyan outbreak sequences is Sabin PV2 – is supported by the evidence shown in the paper, nor evidence from externally available phylogenies. The recombinant parent *may* be a recombinant PV2 – but the proposed parent is not particularly closely related to the query CVA24v sequence in 3Dpol – and it seems equally likely that they both have a separate, but related recombinant parents that are from an unsampled EV-C species. I would not require that the authors agree on this point, but if do believe that if they are going to present any 3D recombination parent as definitive, this needs to be backed up with strong evidence.

As one last note, I tried to compare the phylogenies in Supplementary Figure 5, but I actually the PV colouring is off at least in the 3D plot – the CVA24 sequences (light brown vertical collection of dots) should be near the recombinant PV2 (which should be pale yellow- and the rough topology shape matches) – but instead are shown near dark green (PV1). The coloring for PV1-3 may be off in other panels too (compare the number of sequences coloured dark green, light yellow, and grey between 3B, 3C, and 3D – this should be about the same in each panel, I think?).
