# Supplementary material for: Genomic epidemiology of coxsackievirus A24 variant during the 2024 acute hemorrhagic conjunctivitis outbreak in Coastal Kenya
Source: PLoS Pathog. 2026 Sep 15;22(9):e1014589. doi: 10.1371/journal.ppat.1014589 (PMC13592715; doi:10.1371/journal.ppat.1014589)
Supplement: Supplementary file 5 [file ppat.1014589.s006.docx]

**Reviewers' Comments:**

Reviewer's Responses to Questions

**Comments to the Authors:**

**Please note that one review is uploaded as an attachment.**

**Part I - Summary**
Please use this section to discuss strengths/weaknesses of study, novelty/significance, general execution and scholarship.

Reviewer #1: This manuscript provides a valuable and timely characterization of a coxsackievirus A24 (CVA24v) epidemic detected in Kenya in 2024 through multiple existing surveillance systems and ad hoc investigation of an acute hemorrhagic conjunctivitis outbreak in Mombasa. The generation of a large number of complete genomes significantly strengthens the molecular epidemiology dataset available for this virus, particularly in African contexts where such data are often scarce. The study also contributes to consolidating findings from outbreaks reported in neighboring regions during the same year. Moreover, generating a large number of complete CVA24v genomes (n=245) makes it possible to double the number of available sequences (n=159 as of 1 November 2025) for this virus, which is associated with large-scale outbreaks in tropical and subtropical regions.

However, several aspects of the manuscript require clarification, restructuring, or additional methodological detail to improve readability and scientific rigor.

Response: We thank the reviewer for their positive assessment of our study and for recognising the value of this large African CVA24v genomic data set.

Reviewer #2: Please see uploaded review - I know this is a little less convenient but for this review wanted to include some images. Apologies and thank you!

Response: Not a problem! We thank for the reviewer for their detailed comments and feedback on our paper.

**Part II – Major Issues: Key Experiments Required for Acceptance**
Please use this section to detail the key new experiments or modifications of existing experiments that should be absolutely required to validate study conclusions.

Generally, there should be no more than 3 such required experiments or major modifications for a "Major Revision" recommendation. If more than 3 experiments are necessary to validate the study conclusions, then you are encouraged to recommend "Reject".

Reviewer #1: In my opinion, no additional experiments are necessary to validate study conclusions.

Response: We are thankful for this assessment.

Reviewer #2: To clarify - while I do think the recombination analysis needs to be re-done, I also believe this is absolutely possible and addressable by the authors, and is not a completely new analysis. However it is a modification of an existing 'experiment' (analysis) and thus classified it as such.

Response: We are thankful to the reviewer for the invaluable insights. In the revised manuscript we have re-analysed the data for recombination as per the recommendation.

**Part III – Minor Issues: Editorial and Data Presentation Modifications**
Please use this section for editorial suggestions as well as relatively minor modifications of existing data that would enhance clarity.

Reviewer #1: 1. Inconsistencies between Table 1 and Figure 1
The correspondence between Table 1 and Figure 1 is unclear. According to the title of the table 1, it should indicate the number of samples collected, tested, positive for CVA24v, and sequenced. However, the number of samples actually tested for CVA24v is missing.

For the ResVire surveillance system, Table 1 reports zero samples tested by RT-PCR, whereas Figure 1 indicates qPCR testing. It is not indicated in the legend whether this qPCR was specific for CVA24v. When reading the Materials and Methods section, we understand that two different testing strategies were performed over time (rhinovirus detection with typing until June 2024, then CVA24v-specific RT-PCR). The number of rhinovirus-positive samples that were further screened for CVA24 either by specific qPCR or by VP4-VP2 sequencing should be indicated in the table.
Requested actions:

• Please, indicate in Table 1 the number of samples from the ResVire surveillance actually tested for CVA24v (either by Rhinovirus PCR followed by typing with VP4–VP2 and/or by CVA24-specific qPCR).

Response: We apologize for the confusion. To clarify, the "Number of Samples Collected" column corresponds to the number of samples tested using *any* of the detection methods outlined. In the ResViRe study, 2,742 samples were positive for rhinovirus (RV) via qPCR. Of those, 36 were positive for CVA24v by CVA24v specific qPCR, while 563 were identified as CVA24v by sequencing the VP4/VP2 region. To make this clear to the reader we have added a new column titled "RV positive by qPCR to Table 1."

• Please, clarify in Figure 1 or its legend whether the qPCR assay is specific for CVA24v

Response: We appreciate the reviewer highlighting this important point. While the qPCR used was adapted from a previous published literature (Leveque et al^1^) and designed to be CVA24v-specific, subsequent sequencing revealed cross-reactivity with other enteroviruses, including CVA13 and EV-C99. We have explicitly acknowledged this as a limitation in the Discussion section, emphasizing that confirmatory sequencing remains necessary for definitive CVA24v identification.

• Please, add units to the y-axes (% at left and number at right).

Response: We have updated Figure 1 to included appropriate units on the y-axes.

2. VP1 subgroup definition and phylogenetic support
Lines 174–175. The authors state that the VP1 sequences of CVA24v form 22 distinct subgroups using a threshold of 0.05 nucleotide divergence.
What justifies this threshold?
Are the subgroups supported by reliable bootstrap values? Please indicate bootstrap support values on the branches of the phylogenetic trees, or at least mark branches with strong bootstrap support using an appropriate symbol.

Response: We have clarified our rationale for selecting the 0.05 nucleotide divergence threshold in the Methods section. This threshold was chosen because it produced well-defined clusters that were consistently supported by high bootstrap values (>70%). To demonstrate the reliability of these subgroups, we have now added Bootstrap support values to the relevant branches in the phylogenetic trees.

3. Readibility of the results relative to figures 2 to 4.
The current order of the results presentation makes the narrative difficult to follow, especially since the figures rely on different genomic regions:
- Figure 2A (lines 168-169) - complete genomes
- Figure 3 (lines 174-179) - VP1 sequences
- Figure S1 (lines 178-179) - VP1 sequences (to be indicated in the legend of the figure)
- Figure 2B-2D (lines 187-201) - complete genomes
- Figure 4 (lines 203 ...) - VP1 sequences (this should be indicated in the legend of the figure)
This would improve the readibility to re-organize the Results section to present 1) all phylogenetic analyses based on complete genome (figures 2A-2D), 2) VP1-basedanalyses and subgroups structure and 3) focus on the 2024 sequences.

Response: We have carefully considered the reviewer's suggestion to restructure the Results section to group analyses by genome length (whole genome vs VP1). However, we have respectfully opted to maintain the current flow as it reflects the logical epidemiological progression of the investigation; moving from the global epidemiological context and clade classification down to the high resolution, local dynamics of the 2024 outbreak. To address the reviewer's underlying concern regarding readability, we have explicitly specified whether the analysis utilized whole-genome or partial VP1 sequences in every relevant text section and figure legend. We have also edited the text carefully to improve readability.

4. Terminology regarding the 2024 outbreak

Only three complete genomes originate from patients with AHC symptoms. The remaining sequences come from other surveillance systems without documented conjunctivitis. I suggest that "AHC outreak" should be replaced by "CVA24v outbreak" throughout the manuscript (lines 204, 221, 299, 305, 392).

Response: We agree with this suggestion. Because AHC symptoms were only explicitly documented in a subset of patients, we have replaced the phrase "AHC-outbreak" with "CVA24v outbreak" throughout the manuscript, except when specifically referring to the clinically confirmed AHC outbreak in Mombasa.

5. The statement that CVA24v infections and disease burden are “most pronounced in childhood” (lines 293–295) should be moderated. Two of the four surveillance systems specifically target children, which likely biases sample collection

Response: We agree that the design of our inpatient and outpatient surveillance platforms (which heavily target young children) may introduce an age bias. We have therefore moderated the relevant statement in the Discussion. However, we also note that the community-based ResViRe and health facility out-patient studies that included all age groups found a pattern where most infections were in children. Taken together, we agree that healthcare seeking behaviors and study design do limit our ability to make definitive claims regarding population-level age burden.

**Minor issues**

- According to updated enterovirus nomenclature, there should no longer be a hyphen before the number and the term coxsackie. “CV-A24v” should be replaced with "CVA24v".

Response: We have updated the term "CV-A24v" to "CVA24v" throughout the revised manuscript to comply with the updated enterovirus nomenclature.

- Table 1. The abbreviations AHC and KHDSS should be defined in the legend of the table.

Response: We have defined the abbreviations AHC and KHDSS revised Table 1 legend.

- Figure 3. The red dashed box is not explained in the legend.

Response: The legend for Figure 3 has been updated to indicate that the red dashed box indicates sequences associated with the Asia outbreak while the black dashed box indicates East African sequences.

The manuscript does not reference Figure 4C. If not discussed, it should be removed.

Response: We thank the reviewer for highlighting this. We have now moved Figure 4C to Figure 3C with appropriate text references.

- I assume that CVA24 specific-PCRs were performed retrospectively on samples collected through respiratory or gastrointestinal infection surveillance. This should be indicated in the Materials and Methods section.

Response: We have now explicitly stated in the Methods section that the CVA24v-specific qRT-PCR screening on archival samples was performed retrospectively.

Reviewer #2: (No Response)

Review by Emma Hodcroft

In this paper, “Genomic Epidemiology of Coxsackievirus A24 Variant During the 2024 Acute Hemorrhagic Conjunctivitis Outbreak in Coastal Kenya”, the authors undertake an impressive diagnostic, sequencing, and analysis effort to contribute to a better understanding of the circulation and diversity of CV-A24, particularly in an African setting. Overall, I found this paper well-written and thorough, and a valuable contribution to our understanding of one of many under-studied enteroviruses. In particular, it’s fantastic to see the increase in number of available A24 sequences, and worth noting that the authors have already made these sequences publicly available. The majority of the analyses are appropriate and informative. Most of my comments are minor, though I have one substantive concern with the recombination analysis that I believe requires revisiting in full before publication.

Response: We thank Dr Hodcroft for her thorough and highly constructive review. We particularly appreciate her detailed insights regarding recombination analysis, which prompted us to undertake a much deeper and more robust investigation of the data.

Recombination:

My largest issue with the manuscript is with the recombination analysis. I am not yet convinced that it is clearly shown that there is a potential recombination with poliovirus 1. I believe the analysis needs to be repeated using one of the putative recombinant sequences as the query sequence, including different poliovirus sequences, and including more non-polio EV-C sequences.

Response: We are grateful to the reviewer’s insightful comments on the initial recombination analysis. To address these issues we have completely overhauled the recombination analysis and present a revised version which we believe is clearer and more compelling.

In particular, we have significantly expanded our comparative data set, now analyzing our 245 new sequences against a global data set of 2,418 complete or near complete genomes that includes Poliovirus 1, 2 and 3, as well as a wide array of non-polio *Enterovirus coxsackiepol* (EV-C) sequences. In addition, following the reviewer’s recommendations, we inverted the similarity plot to use a Kenyan CVA24v sequence (GenBank accession number: PX659173) as the query sequence. This revised analysis identified interspecies recombination event between the 2024 East African Outbreak (Kenya Malawi and Mayotte) and Sabin PV2 sequences from West Africa. These updated findings, along with improved plots incorporating polioviruses and non-polio EV-Cs, have now been integrated into Results, Discussion and Figure 6/7 of the revised manuscript.

In Figure 6 A, it is unclear what the query sequences is (the similarity against which the other sequences are plotted). I would have expected it to be potentially one of the Kenya 2024 sequences, but Kenya_2024 is instead listed as one of the references. When the legend refers to ‘Kenya_2024’ is this one example sequence or a consensus – if the latter how was it generated? (Similar questions for the other sequences included.) What is the cluster of the references included (e.g. C1/1/24, C1/2/24, C1/3/24)? How were the particular polio sequences included chosen?

Response: In our revised analysis one of the Kenya sequences (GenBank accession number: PX659173) was used as the query sequence. The "Kenya_2024 " is consensus sequence of all the Kenya 2024 sequences (n=245), or "Mayotte_2024" refers to all Mayotte sequences (n=8). The consensus is generate by an in inhouse R script by collapsing defined sequence groups into majority-rule consensus sequences, which are then compared to the query sequences across a 200 nt sliding genomic window. In this analysis all Kenyan and East Africa sequences (irrespective of their C1/1/24, C1/2/24, or C1/3/24 classification) were grouped into a single consensus since no substantial sequence differences were observed among the three groups overall (similarity index ranging from 0.997 to 1.0), they were analysed as a single group.

Figure 6 A shows a drop in similarity between the query (as well as other A24v viruses) and Kenya_2024, Uganda_2017, Malawi_2024, and Mayotte_2024, at just after 5100bp, which could indicate recombination. These sequences drop to the same level of similarity as the included poliovirus sequences. Importantly this does not necessarily indicate that the Kenya/Uganda/Malawi/Mayotte sequences are similar to the polio sequences – it rather indicates that they and the polio sequences are roughly equally divergent from the query. This plot should really be inverted – traditionally one would make the query the suspected recombinant sequence, meaning similarity is then relative to the suspected recombinant. This is important as the graph shows distance from the query sequence, not similarity to the other references – 2 sequences could both be 20% distant from the query, but not be identical to each other (indeed, they could be very diverged). Thus, to show that the putative recombinant sequences are very similar to the poliovirus sequences, one needs to invert the plotting.

Equally importantly, no other non-polio EV-Cs are included in the graph. This is critical, as it’s impossible to determine if the distance between these four sequences/sequence groups (Kenya/Uganda/Malawi/Mayotte) and the polio sequences is close, or just closer relative to the query (perhaps any other EV-C sequence would be equally ‘distant’ from the query if others were plotted?). After inverting the graph, including other non-polio EV-Cs will help to clarify if there are any closer putative parents, or indeed if other EV-C sequences are as close (or distant) from the query as the polio sequences.

Response: The plot has been reverted and results discussed above

Notably the Malawi_2024 sequence has a sharp increase in similarity compared to the others (Kenya/Uganda/Mayotte) shortly after the drop in similarity – why is this? Also, there’s a small gap visible in the graph just before this – what is this?

Response: The Malawi sequence was very gappy/failed sequencing, mainly in the 3Dpol gene. This resulted in the drop and gap in the graph.

In such plots, it’s also important to remember that EVs are relatively similar (particularly within type), and that similarity varies greatly across the genome – with some areas being more conserved than others. Finding diversity of 70-80% (line 260) between EV-Cs is not necessarily unusual, particularly in this region.

The authors then move directly to an RDP5 analysis, but again seemingly only include polio sequences (and only poliovirus 1 is shown in Fig 6 B) in their analysis. (Please label all potential parents directly in Fig 6 B – it’s not clear to me why there are 3 lines.) This means it is not possible to conclude that another EV-C, or indeed that PV-2 or 3, could not be the recombination partner. The same is true for the phylogenetic trees made with sections of the genome (Figure 7), which only include poliovirus sequences. (In this figure it is also impossible to see the PV-1 sequence(s?) and thus difficult to assess whether PV-1 may group more closely than PV-2 or PV-3).

Response: To address this point we have now repeated the recombination analysis using 245 sequences generated in this study and expanded global dataset of n=2,418 sequences including poliovirus 1, 2 and 3, as well as non-polio EV-C sequences. One of the Kenya sequence (GenBank accession number: PX659173) was used as the query sequence. It was clear from this analysis that CVA24v exhibited a recombination signal with other EV-C virus i.e., polioviruses 1 and 2. Figure 6 shows where a recombination signal was detected between CVA24v and PV2 spanning position 6080 - 7341 (ungapped alignment and 6418 – 7627 in gapped equivalent). This signal was seen in the East African (Kenya-2024, Uganda-2017, Malawi-2024, and Mayotte-2024) sequences. These findings have been incorporated into the Results and Discussion sections of the revised manuscript.

Beyond the need to include more EV-C sequences, there is a further complication that is worth considering when interpreting the polio sequences already included in the analysis. It’s important to remember that poliovirus sequences themselves are often recombinants. In particular, for the polio sequences used in Fig 6 A), our independent pre-existing analysis of EV-C genomes shows there’s a good chance these sequences are recombinant themselves in the 3D gene. This graph (from existing internal work) shows a tangle-gram of VP1 vs 3D, coloring sequences by their VP1 type, and shows that in the 3D gene, many poliovirus 1, 2, and 3 sequences split in their associations between VP1 and 3D.

Response: We agree with reviewer. Importantly, there is clear evidence suggesting that some poliovirus genomes may themselves be recombinant, as indicated by the phylogenetic incongruence observed in Figure 7 (See below) and Supplementary Figure 5.

Highlighting the specific sequences used in Fig 6 A) (highlighted in blue) it’s possible to see that the PV1 and PV3 sequences follow the minority of the clade and split to the lower part of the tree, close to part of PV2 (in the case of PV1, actually interleaving with PV2).

Further, if we assume the A24v recombination partner is most likely derived from a vaccine-derived polio virus (originally from the Sabin vaccines), since endemic wild polio was last detected in 1984, we would expect the 3D segment of A24v to be closer to the Sabin genome. However, the Sabin genomes (highlighted in yellow) are instead quite distant from the included poliovirus sequences that are ‘similar’ to the A24v sequences. Thus it seems very possible that all of the included poliovirus sequence may be recombinant themselves. The recombination partner for these poliovirus sequences and the A24v sequences could then perhaps be an unknown EV-C (or multiple such viruses, relatively closely related) and have recombined with these viruses separately. Alternatively, perhaps the original recombination occurred in PV2, and this recombinant has then recombined again with A24v (but importantly, it doesn’t seem to be the original Sabin2 3D which is the recombination partner).

Response: The repeat phylogenetic analysis identified inter-species recombination between 2024 Kenya CVA24v sequences and Sabin PV2 sequences, with CVA24v serving as the major parent and PV2 as the minor parent. The 3D sequences from the 2024 East African outbreak( Kenya, Mayotte, Malawi and 2017 Uganda) clustered closely to Sabin PV2 sequences from West Africa. These findings have been incorporated into the Results and Discussion sections of the revised manuscript.

Additionally, Figure 6 A is quite difficult to read – the colors are not distinguishable and the lines often obscure each other. I would suggest showing only the most vital and convincing sequences or sequence-groups (perhaps 10) on the main-text figure and then including more plots as supplemental figures, showing similarity to other sequences or groups of sequences. And in line with my suggestion above, it would be good to include other non-polio EV-C viruses in supplementary figs (e.g. Fig S4) – and ensure PV1 sequences are visible (and potentially include more of them?)

Response: This has been corrected in the manuscript.

Finally, on a related note, if such a recombination is present, this will also influence the estimate of the MRCA for any whole-genome datasets that include both recombinant and non-recombinant sequences (estimating the MRCA for only recombinant sequences should be ok). Thus for the global MRCA estimates (section starting line 187) the authors should consider masking the recombinant region or consider alternative ways to address this.

Response: This is good point. Hence, to account for the potential effects of recombination on molecular clock inference, we reran the BEAST analysis after masking the suspected recombinant region and re-estimated the MRCA.

**Smaller issues:**

- Given the confusing naming of Enteroviruses in general, it would be worth clarifying in the introduction that A24 is an EV-C (the name unfortunately could convey it is an EV-A)

Response: We have updated the introduction to clearly state that CA24v is a member of *Enterovirus coxsackiepol* species (formerly EV-C).

- CV-A24v is a variant or subclade of CV-A24, but this relationship isn’t made very clear in the paper (“caused by Coxsackievirus A24 variant” could be read as just being the A24 variant of the larger EV-C group), and would be worth being a little more explicit about. It would be useful to hear what proportion of A24 is A24v and if there’s any general information about how the subclade is defined (e.g. by specific mutations).

Response: CVA24v is a genetically and epidemiologically distinct lineage of CV-A24 within the species *Enterovirus coxsackiepol* (formerly *Enterovirus C*). Coxsackievirus A24 (CVA24) was first isolated in South Africa in 1951, but was not associated with disease in humans. CVA24v, which is a pathogenic variant of CVA24 (CVA24v) was identified in Singapore in 1970 and has globally associated with acute hemorrhagic conjunctivitis (AHC) outbreaks. Since its emergence, nearly all documented large-scale AHC epidemics have been attributed to CVA24v strains, while classical/non-variant CV-A24 strains are now rarely reported in contemporary surveillance data sets. In our study, all detected viruses clustered within the CVA24v lineage, and no prototype/classical CV-A24 strains were identified.

- In the results (line 161) it’s mentioned there are 8 identified genotypes of A24v– it would be helpful to mention this earlier, in the introduction.

Response: A paragraph has been added to the Introduction describing CVA24v genotypes and outlining the basis for their classification.

- In Table 1, it was a little hard to match up the numbers with those mentioned in the text. For example, WG sequencing was attempted on 333 samples (line 148), presumably out of 178+602 (Table 1) positives. Was the reduced number primarily because of the CT threshold mentioned, or due to false-positives meaning some of the positive samples were actually other EVs (cross-reactivity, mentioned on line 386-388), or other reasons?

Response: We have now clarified in the Results section that the reduction from PCR-positive samples to successfully sequenced whole genomes was primarily due to generic quality constraints (e.g., high Ct (low viral load) and poor RNA quality). We applied a strict cut-off of Ct <30.0 to increase genome recovery yields. Additionally, a limited number of samples were dropped off due to assay cross-reactivity e.g., identification a CV-A13 and EV-C99 sequences in some samples. We have clarified this in the manuscript.

- In line 111-2 it’s mentioned that sequenced samples were combined with all publicly A24v available data – is this all publicly available whole-genomes, or any length? How did the authors identify public sequences as being A24v rather than A24?

Response: We included all complete or near complete CVA24v genome sequences (> 6500 bp, n=159, as of 01 November 2025) available on GenBank. The downloaded genomes were identified as CVA24v using an online Enterovirus Genotyping Tool. In addition, we downloaded and included 567 available VP1 sequences in assessing the clustering patterns and classification among the CVA24v genotype IV (G-IV) sequences. This has been updated in the manuscript. Further, 2,173 complete or near complete *Enterovirus Coxsackiepol* genome sequences were included in the recombination analysis

- If possible it would be interesting to dig a little more into the amino-acids under selection (section starting line 222) – are any of these surface exposed, or suspected/confirmed epitopes? Is anything else known about these locations?

Response: While determining the biological relevance of these positively selected sites is a fascinating prospect, there is currently limited functional or structural mapping data available for these specific residues in CVA24v. To avoid over-speculating these findings in the absence of the supporting experimental or structural data we have maintained a conservative interpretation. Further, we have explicitly highlighted that this is an important area for future investigation to explore the structural context and potential epitope associations of these residues.

**Minor comments/typos:**

- Line 90 – ‘viral capsid (VP) protein’ should probably be ‘viral capsid protein (VP)’

Response: This has been corrected in the manuscript.

- Line 107-8 – It would be good to reference Fig S5 here so that readers can orient themselves with the geography

Response: S5 Fig has now been cited in the text and has been renumbered as S1 Fig throughout the manuscript.

- Line 112 – Specify if only WGS publicly available genomes were included (or also partial genomes)

Response: We have clarified that all 159 publicly available CVA24v sequences (as of 01 November 2025) used were complete or near complete genomes (>6500 bp), while the 567 VP1 partial sequences analyzed separately for sub-grouping.

- Line 113 – ‘which largely focused descriptive’ > ‘which largely focused on descriptive’

Response: This has been corrected in the manuscript.

- Line 125 – ‘peak CV-A24v was observed’ – perhaps ‘samples’ or ‘cases’ is missing?

Response: This has been corrected in the manuscript.

- Line 137 – ‘were more frequently in infants’ > ‘were more frequent in infants’

Response: This has been corrected in the manuscript.

- Line 138-140 – Is it possible to add these locations to the map in Fig S5?

Response: Locations have been added on the map

- Line 168 – ‘revealed viruses’ > ‘revealed that viruses’

Response: This has been corrected in the manuscript.

- Line 172 – Does ‘dereplicated’ mean ‘deduplicated’?

Response: Yes.

- Line 182 – ‘they close to the’ > ‘they are close to the’

Response: This has been corrected in the manuscript.

- Line 366 – Closing parenthesis missing after ‘South America’

Response: This has been corrected in the manuscript.

- Figure 1 – Please add labels to the axes and indicate more clearly which corresponds to the line & which to the bars. In addition, the red and green are sometimes hard to distinguish on some screens – and very difficult to distinguish for red/green colour blind.

Response: This has been corrected in the manuscript.

- Figure 2, the slope is given as 5.17e-03, but in the text the rate is given as 5.37x10^-3 (line 191) – possible typo?

Response: This has been corrected in the manuscript.

- Figure 2 E – The colours used in the world map are a little contrary to norms – usually yellow/red are ‘hotter’ and therefore ‘more’ and blue represents ‘less’

Response: We have adjusted the colouring of the Figure 2E world map to reflect the standard data visualization norms (red to show highest and blue to show the lowest).

- Figure 3 – Please add labels to the axes

Response: This has been corrected in the manuscript.

- Figure 3 B – The text says there are 22 distinct sub-groups (line 174), but only 17 are shown in the figure. Among the missing are C12, C14, C15, C21

Response: This has been corrected in the manuscript. 6 sub-groups were excluded as they presented as singletons.

- Figure S1 B – the labels are cutoff on the right, one can’t see the last letter of the clustering groups

Response: The Figure has been corrected and changed to Figure S2B.
