# Supplementary material for: Genomic epidemiology of coxsackievirus A24 variant during the 2024 acute hemorrhagic conjunctivitis outbreak in Coastal Kenya
Source: PLoS Pathog. 2026 Sep 15;22(9):e1014589. doi: 10.1371/journal.ppat.1014589 (PMC13592715; doi:10.1371/journal.ppat.1014589)
Supplement: Peer review history

# Peer review: Decision Letter 0

**DOI:** 10.1371/journal.ppat.1014589.r001

**Authors:** Ronald Swanstrom

20 Apr 2026

Genomic Epidemiology of Coxsackievirus A24 Variant During the 2024 Acute Hemorrhagic Conjunctivitis Outbreak in Coastal Kenya

PLOS Pathogens

Dear Dr. Morobe,

Thank you for submitting your manuscript to PLOS Pathogens. After careful consideration, we feel that it has merit but does not fully meet PLOS Pathogens's publication criteria as it currently stands. Therefore, we invite you to submit a revised version of the manuscript that addresses the points raised during the review process. You will note that Reviewer 2 has provided a detailed description with examples of the issue of recombination and the extent to which it confounds your analysis. This is a major point that will need to be resolved. Those comments are in an attachment; please let me know if the attachment is not included.

Please submit your revised manuscript by Jun 19 2026 11:59PM. If you will need more time than this to complete your revisions, please reply to this message or contact the journal office at plospathogens@plos.org. When you're ready to submit your revision, log on to https://www.editorialmanager.com/ppathogens/ and select the 'Submissions Needing Revision' folder to locate your manuscript file.

Please include the following items when submitting your revised manuscript:

* A letter that responds to each point raised by the editor and reviewer(s). You should upload this letter as a separate file labeled 'Response to Reviewers'. This file does not need to include responses to any formatting updates and technical items listed in the 'Journal Requirements' section below.

* A marked-up copy of your manuscript that highlights changes made to the original version. You should upload this as a separate file labeled 'Revised Manuscript with Track Changes'.

* An unmarked version of your revised paper without tracked changes. You should upload this as a separate file labeled 'Manuscript'.

If you would like to make changes to your financial disclosure, competing interests statement, or data availability statement, please make these updates within the submission form at the time of resubmission. Guidelines for resubmitting your figure files are available below the reviewer comments at the end of this letter.

We look forward to receiving your revised manuscript.

Kind regards,

Ronald Swanstrom

Section Editor

PLOS Pathogens

Ronald Swanstrom

Section Editor

PLOS Pathogens

Editor-in-Chief

PLOS Pathogens

orcid.org/0000-0003-2946-9497

Michael Malim

Editor-in-Chief

PLOS Pathogens

orcid.org/0000-0002-7699-2064

**Journal Requirements:**

1) We do not publish any copyright or trademark symbols that usually accompany proprietary names, eg ©,  ®, or TM  (e.g. next to drug or reagent names). Therefore please remove all instances of trademark/copyright symbols throughout the text, including:

- ® on page: 22.

2) Thank you for including an Ethics Statement for your study. Please include:

i) The full name(s) of the Institutional Review Board(s) or Ethics Committee(s)

ii) Please state whether the consent obtained is verbal or written

3) Please amend your detailed Financial Disclosure statement. This is published with the article. It must therefore be completed in full sentences and contain the exact wording you wish to be published.

1) State the initials, alongside each funding source, of each author to receive each grant. For example: "This work was supported by the National Institutes of Health (####### to AM; ###### to CJ) and the National Science Foundation (###### to AM)."

2) State what role the funders took in the study. If the funders had no role in your study, please state: "The funders had no role in study design, data collection and analysis, decision to publish, or preparation of the manuscript."

4) Some material included in your submission may be copyrighted. According to PLOS’s copyright policy, authors who use figures or other material (e.g., graphics, clipart, maps) from another author or copyright holder must demonstrate or obtain permission to publish this material under the Creative Commons Attribution 4.0 International (CC BY 4.0) License used by PLOS journals. Please closely review the details of PLOS’s copyright requirements here: PLOS Licenses and Copyright. If you need to request permissions from a copyright holder, you may use PLOS's Copyright Content Permission form.

Please respond directly to this email and provide any known details concerning your material's license terms and permissions required for reuse, even if you have not yet obtained copyright permissions or are unsure of your material's copyright compatibility. Once you have responded and addressed all other outstanding technical requirements, you may resubmit your manuscript within Editorial Manager.

Potential Copyright Issues:

Figures 2E, S5 :please (a) provide a direct link to the base layer of the map (i.e., the country or region border shape) and ensure this is also included in the figure legend; and (b) provide a link to the terms of use / license information for the base layer image or shapefile. We cannot publish proprietary or copyrighted maps (e.g. Google Maps, Mapquest) and the terms of use for your map base layer must be compatible with our CC BY 4.0 license.

Note: if you created the map in a software program like R or ArcGIS, please locate and indicate the source of the basemap shapefile onto which data has been plotted.

If your map was obtained from a copyrighted source please amend the figure so that the base map used is from an openly available source. Alternatively, please provide explicit written permission from the copyright holder granting you the right to publish the material under our CC BY 4.0 license.

If you are unsure whether you can use a map or not, please do reach out and we will be able to help you. The following websites are good examples of where you can source open access or public domain maps:

* U.S. Geological Survey (USGS) - All maps are in the public domain. (http://www.usgs.gov)

* PlaniGlobe - All maps are published under a Creative Commons license so please cite “PlaniGlobe, http://www.planiglobe.com, CC BY 2.0” in the image credit after the caption. (http://www.planiglobe.com/?lang=enl)

* Natural Earth - All maps are public domain. (http://www.naturalearthdata.com/about/terms-of-use/)".

Note: If the reviewer comments include a recommendation to cite specific previously published works, please review and evaluate these publications to determine whether they are relevant and should be cited. There is no requirement to cite these works unless the editor has indicated otherwise.

**Reviewers' Comments:**

Reviewer's Responses to Questions

**Comments to the Authors:**

**Please note that one review is uploaded as an attachment.**

**Part I - Summary**

Please use this section to discuss strengths/weaknesses of study, novelty/significance, general execution and scholarship.

Reviewer #1: This manuscript provides a valuable and timely characterization of a coxsackievirus A24 (CVA24v) epidemic detected in Kenya in 2024 through multiple existing surveillance systems and ah hoc investigation of an acute hemorrhagic conjonctivitis outbreak in Mombasa.

The generation of a large number of complete genomes significantly strengthens the molecular epidemiology dataset available for this virus, particularly in African contexts where such data are often scarce. The study also contributes to consolidating findings from outbreaks reported in neighboring regions during the same year. Moreover, generating a large number of complete CVA24v genomes (n=245) makes it possible to double the number of available sequences (n=159 as of 1 November 2025) for this virus, which is associated with large-scale outbreaks in tropical and subtropical regions.

However, several aspects of the manuscript require clarification, restructuring, or additional methodological detail to improve readability and scientific rigor.

Reviewer #2: Please see uploaded review - I know this is a little less convenient but for this review wanted to include some images. Apologies and thank you!

**********

**Part II – Major Issues: Key Experiments Required for Acceptance**

Please use this section to detail the key new experiments or modifications of existing experiments that should be absolutely required to validate study conclusions.

Generally, there should be no more than 3 such required experiments or major modifications for a "Major Revision" recommendation. If more than 3 experiments are necessary to validate the study conclusions, then you are encouraged to recommend "Reject".

Reviewer #1: In my opinion, no additional experiments are necessary to validate study conclusions.

Reviewer #2: To clarify - while I do think the recombination analysis needs to be re-done, I also believe this is absolutely possible and addressable by the authors, and is not a completely new analysis. However it is a modification of an existing 'experiment' (analysis) and thus classified it as such.

**********

**Part III – Minor Issues: Editorial and Data Presentation Modifications**

Please use this section for editorial suggestions as well as relatively minor modifications of existing data that would enhance clarity.

Reviewer #1: 1. Inconsistencies between Table 1 and Figure 1

The correspondence between Table 1 and Figure 1 is unclear. According to the title of the table 1, it should indicate the number of samples collected, tested, positive for CVA24v, and sequenced. However, the number of samples actually tested for CVA24v is missing.

For the ResVire surveillance system, Table 1 reports zero samples tested by RT-PCR, whereas Figure 1 indicates qPCR testing. It is not indicated in the legend whether this qPCR was specific for CVA24v. When reading the Materials and Methods section, we understand that two different testing strategies were performed over time (rhinovirus detection with typing until June 2024, then CVA24v-specific RT-PCR). The number of rhinovirus-positive samples that were further screened for CVA24 either by specific qPCR or by VP4-VP2 sequencing should be indicated in the table.

Requested actions:

• Please, indicate in Table 1 the number of samples from the ResVire surveillance actually tested for CVA24v (either by Rhinovirus PCR followed by typing with VP4–VP2 and/or by CVA24-specific qPCR).

• Please, clarify in Figure 1 or its legend whether the qPCR assay is specific for CVA24v

• Please, add units to the y-axes (% at left and number at right).

2. VP1 subgroup definition and phylogenetic support

Lines 174–175. The authors state that the VP1 sequences of CVA24v form 22 distinct subgroups using a threshold of 0.05 nucleotide divergence.

What justifies this threshold?

Are the subgroups supported by reliable bootstrap values? Please indicate bootstrap support values on the branches of the phylogenetic trees, or at least mark branches with strong bootstrap support using an appropriate symbol.

3. Readibility of the results relative to figures 2 to 4.

The current order of the results presentation makes the narrative difficult to follow, especially since the figures rely on different genomic regions:

- Figure 2A (lines 168-169) - complete genomes

- Figure 3 (lines 174-179) - VP1 sequences

- Figure S1 (lines 178-179) - VP1 sequences (to be indicated in the legend of the figure)

- Figure 2B-2D (lines 187-201) - complete genomes

- Figure 4 (lines 203 ...) - VP1 sequences (this should be indicated in the legend of the figure)

This would improve the readibility to re-organize the Results section to present 1) all phylogenetic analyses based on complete genome (figures 2A-2D), 2) VP1-based analyses and subgroups structure and 3) focus on the 2024 sequences.

4. Terminology regarding the 2024 outbreak

Only three complete genomes originate from patients with AHC symptoms. The remaining sequences come from other surveillance systems without documented conjunctivitis. I suggest that "AHC outreak" should be replaced by "CVA24v outbreak" throughout the manuscript (lines 204, 221, 299, 305, 392).

5. The statement that CVA24v infections and disease burden are “most pronounced in childhood” (lines 293–295) should be moderated. Two of the four surveillance systems specifically target children, which likely biases sample collection

Minor issues

- According to updated enterovirus nomenclature, there should no longer be a hyphen before the number and the term coxsackie. “CV-A24v” should be replaced with "CVA24v".

- Table 1. The abbreviations AHC and KHDSS should be defined in the legend of the table.

- Figure 3. The red dashed box is not explained in the legend.

The manuscript does not reference Figure 4C. If not discussed, it should be removed.

- I assume that CVA24 specific-PCRs were performed retrospectively on samples collected through respiratory or gastrointestinal infection surveillance. This should be indicated in the Materials and Methods section.

Reviewer #2: (No Response)

**********

PLOS authors have the option to publish the peer review history of their article (what does this mean?). If published, this will include your full peer review and any attached files.

If you choose “no”, your identity will remain anonymous but your review may still be made public.

**Do you want your identity to be public for this peer review?**  For information about this choice, including consent withdrawal, please see our Privacy Policy.

Reviewer #1: No

Reviewer #2: **Yes:** Emma B Hodcroft

[NOTE: If reviewer comments were submitted as an attachment file, they will be attached to this email and accessible via the submission site. Please log into your account, locate the manuscript record, and check for the action link "View Attachments". If this link does not appear, there are no attachment files.]

**Figure resubmission:**

**Reproducibility:**

Attachment Submitted filename: Hodcroft-Review.pdf

# Author comment: Author response to Decision Letter 1

**DOI:** 10.1371/journal.ppat.1014589.r002

22 Jun 2026

Attachment Submitted filename: Response to Reviewers_18062026.docx

# Peer review: Decision Letter 1

**DOI:** 10.1371/journal.ppat.1014589.r003

**Authors:** Ronald Swanstrom

22 Jul 2026

PPATHOGENS-D-26-00243R1

Genomic Epidemiology of Coxsackievirus A24 Variant During the 2024 Acute Hemorrhagic Conjunctivitis Outbreak in Coastal Kenya

PLOS Pathogens

Dear Dr. Morobe,

Thank you for submitting your manuscript to PLOS Pathogens. After careful consideration, we feel that it has merit but still does not fully meet PLOS Pathogens's publication criteria as it currently stands. Therefore, we invite you to submit a revised version of the manuscript that addresses the points raised during the review process.

I will note this is an unusual situation in which the reviewer has an extremely high level of expertise and familiarity with your specific study. I agree with her that it would be unfortunate to publish a conclusion that could be wrong and would lead to misunderstanding, especially in the public health field. I think this is a sincere concern on her part, and we all have a vested interest in making our interpretations as defensible as possible. The reviewer has offered to help directly if you feel you need better guidance on the part of your study that is in question. It is your choice but again, given the importance of the conclusion, it is important that you make the analysis as strong as possible. If you would like to work directly with the reviewer on this point I would be happy to mediate that interaction. You can send me an email and I will work to get you two in communication (my email is risunc@med.unc.edu). While this is an unusual situation, it does happen where we use the peer review process not just to judge but to do whatever we can to improve the outcome.

Please submit your revised manuscript by Sep 20 2026 11:59PM. If you will need more time than this to complete your revisions, please reply to this message or contact the journal office at plospathogens@plos.org. When you're ready to submit your revision, log on to https://www.editorialmanager.com/ppathogens/ and select the 'Submissions Needing Revision' folder to locate your manuscript file.

Please include the following items when submitting your revised manuscript:

* A letter that responds to each point raised by the editor and reviewer(s). You should upload this letter as a separate file labeled 'Response to Reviewers'. This file does not need to include responses to any formatting updates and technical items listed in the 'Journal Requirements' section below.

* A marked-up copy of your manuscript that highlights changes made to the original version. You should upload this as a separate file labeled 'Revised Manuscript with Track Changes'.

* An unmarked version of your revised paper without tracked changes. You should upload this as a separate file labeled 'Manuscript'.

If you would like to make changes to your financial disclosure, competing interests statement, or data availability statement, please make these updates within the submission form at the time of resubmission. Guidelines for resubmitting your figure files are available below the reviewer comments at the end of this letter.

As the corresponding author, your ORCID iD is verified in the submission system and will appear in the published article. PLOS supports the use of ORCID, and we encourage all coauthors to register for an ORCID iD and use it as well. Please encourage your coauthors to verify their ORCID iD within the submission system before final acceptance, as unverified ORCID iDs will not appear in the published article. Only the individual author can complete the verification step; PLOS staff cannot verify ORCID iDs on behalf of authors.

We look forward to receiving your revised manuscript.

Kind regards,

Ronald Swanstrom

Section Editor

PLOS Pathogens

Ronald Swanstrom

Section Editor

PLOS Pathogens

Sumita Bhaduri-McIntosh

Editor-in-Chief

PLOS Pathogens

orcid.org/0000-0003-2946-9497

Michael Malim

Editor-in-Chief

PLOS Pathogens

orcid.org/0000-0002-7699-2064

**Reviewers' Comments:**

Reviewer's Responses to Questions

**Part I - Summary**

Please use this section to discuss strengths/weaknesses of study, novelty/significance, general execution and scholarship.

Reviewer #2: Please see attached review

**********

**Part II – Major Issues: Key Experiments Required for Acceptance**

Please use this section to detail the key new experiments or modifications of existing experiments that should be absolutely required to validate study conclusions.

Generally, there should be no more than 3 such required experiments or major modifications for a "Major Revision" recommendation. If more than 3 experiments are necessary to validate the study conclusions, then you are encouraged to recommend "Reject".

Reviewer #2: Please see attached review

**********

**Part III – Minor Issues: Editorial and Data Presentation Modifications**

Please use this section for editorial suggestions as well as relatively minor modifications of existing data that would enhance clarity.

Reviewer #2: Please see attached review

**********

PLOS authors have the option to publish the peer review history of their article (what does this mean?). If published, this will include your full peer review and any attached files.

If you choose “no”, your identity will remain anonymous but your review may still be made public.

**Do you want your identity to be public for this peer review?**  For information about this choice, including consent withdrawal, please see our Privacy Policy.

Reviewer #2: **Yes:** Emma Hodcroft

[NOTE: If reviewer comments were submitted as an attachment file, they will be attached to this email and accessible via the submission site. Please log into your account, locate the manuscript record, and check for the action link "View Attachments". If this link does not appear, there are no attachment files.]

**Figure resubmission:**

**Reproducibility:**

To enhance the reproducibility of your results, we recommend that authors of applicable studies deposit laboratory protocols in protocols.io, where a protocol can be assigned its own identifier (DOI) such that it can be cited independently in the future. Additionally, PLOS ONE offers an option to publish peer-reviewed clinical study protocols. Read more information on sharing protocols at https://plos.org/protocols?utm_medium=editorial-email&utm_source=authorletters&utm_campaign=protocols

Attachment Submitted filename: Hodcroft-RevisionReview.docx

# Author comment: Author response to Decision Letter 2

**DOI:** 10.1371/journal.ppat.1014589.r004

18 Aug 2026

Attachment Submitted filename: Response to reviewer_18082026.docx

# Editor report: Decision Letter 2

**DOI:** 10.1371/journal.ppat.1014589.r005

**Authors:** Ronald Swanstrom

1 Sep 2026

Dear Mr Morobe,

We are pleased to inform that your manuscript, "Genomic Epidemiology of Coxsackievirus A24 Variant During the 2024 Acute Hemorrhagic Conjunctivitis Outbreak in Coastal Kenya", has been editorially accepted for publication at PLOS Pathogens.

Before your manuscript can be formally accepted and sent to production, you will need to complete our formatting changes, which you will receive by email within a week. Please note that your manuscript will not be scheduled for publication until you have made the required changes.

IMPORTANT NOTES

(1) Copyediting and Proofreading: The corresponding author will receive a typeset proof for review, to ensure errors have not been introduced during production. Please review the PDF proof of your manuscript carefully, as this is the last chance to correct any errors. Please note that major changes, or those which affect the scientific understanding of the work, will likely cause delays to the publication date of your manuscript.

(2) Appropriate Figure Files: Please remove all name and figure # text from your figure files. Please also take this time to check that your figures are of high resolution, which will improve the readbility of your figures and help expedite your manuscript's publication. Please note that figures must have been originally created at 300dpi or higher. Do not manually increase the resolution of your files. For instructions on how to properly obtain high quality images, please review our Figure Guidelines, with examples at: http://journals.plos.org/plospathogens/s/figures.

We encourage you to use PACE (http://pace.apexcovantage.com/), a figure-checking tool which helps authors ensure all figures meet PLOS requirements so that figures can publish in the highest quality. PACE is a digital diagnostic and conversion tool for figure files. It will provide information about any failed check(s) and, if able, will automatically convert the figure into an acceptable file that passes quality checks.

To use PACE, you must first register as a user. Then, login and navigate to the UPLOAD tab, where you will find detailed instructions on how to use the tool.

(3) Striking Image: Please upload a striking still image to accompany your article if one is available (you can include a new image or an existing one from within your manuscript). Should your paper be accepted, this image will be considered for our monthly issue image and may also appear on our website to feature your article. Please upload this as a separate file, selecting "striking image" as the file type upon upload. Please also include a separate "Other" file with a caption, including credits and any potential copyright information. Please do not include the caption in the main article file. If your image is from someone other than yourself, please ensure that the artist has read and agreed to the terms and conditions of the Creative Commons Attribution License at http://journals.plos.org/plospathogens/s/content-license. Please note that PLOS cannot publish copyrighted images.

(4) Press Release or Related Media: If you or your institution will be preparing press materials for this manuscript, please inform our press team in advance at plospathogens@plos.org as soon as possible. We ask that you contact us within one week to plan ahead of our fast Production schedule. If you need to know your paper's publication date for related media purposes, you must coordinate with our press team, and your manuscript will remain under a strict press embargo until the publication date and time.

(5) Update your Profile Information: Now that your manuscript has been provisionally accepted, please log into Editorial Manager and update your profile, if needed. Go to http://www.editorialmanager.com/ppathogens, log in, and click on the "Update My Information" link at the top of the page. Please update your user information to ensure an efficient production and billing process.

(6) LaTeX users only: Our staff will ask you to upload a TEX file in addition to the PDF before the paper can be sent to typesetting, so please carefully review our Latex Guidelines http://journals.plos.org/plospathogens/s/latex in the meantime.

Thank you again for supporting Open Access publishing; we are looking forward to publishing your work in PLOS Pathogens.

Best regards,

Emma Hodcroft

Guest Editor

PLOS Pathogens

Ronald Swanstrom

Section Editor

PLOS Pathogens

Ronald Swanstrom

Section Editor

PLOS Pathogens

Dominique Soldati-Favre

Editor-in-Chief

PLOS Pathogens

***********************************************************

# Editor report: Acceptance letter

**DOI:** 10.1371/journal.ppat.1014589.r006

**Authors:** Ronald Swanstrom

Dear Mr Morobe,

We are delighted to inform you that your manuscript, "Genomic Epidemiology of Coxsackievirus A24 Variant During the 2024 Acute Hemorrhagic Conjunctivitis Outbreak in Coastal Kenya," has been formally accepted for publication in PLOS Pathogens.

We have now passed your article onto the PLOS Production Department who will complete the rest of the pre-publication process. All authors will receive a confirmation email upon publication.

Thank you again for supporting open-access publishing; we are looking forward to publishing your work in PLOS Pathogens.

Best regards,

Dominique Soldati-Favre

Editor-in-Chief

PLOS Pathogens
